# Are Hippocampal Hypoperfusion and ATP Depletion Prime Movers in the Genesis of Alzheimer’s Disease? A Review of Recent Pertinent Observations from Molecular Biology

**DOI:** 10.3390/ijms26157328

**Published:** 2025-07-29

**Authors:** Valerie Walker

**Affiliations:** Department of Clinical Biochemistry, University Hospital Southampton NHS Foundation Trust, Southampton General Hospital, Southampton SO16 6YD, UK; valerie.walker@uhs.nhs.uk; Tel.: +44-2381206436

**Keywords:** ATP biosensors, malate aspartate shuttle, glutamate/GABA/glutamine cycle, axonal transport, vasospasm, cerebral arterial perfusion, mitochondrial-derived peptides, membrane phospholipids

## Abstract

Alzheimer’s dementia (AD) is a disease of the ageing brain. It begins in the hippocampal region with the epicentre in the entorhinal cortex, then gradually extends into adjacent brain areas involved in memory and cognition. The events which initiate the damage are unknown and under intense investigation. Localization to the hippocampus can now be explained by anatomical features of the blood vessels supplying this region. Blood supply and hence oxygen delivery to the area are jeopardized by poor flow through narrowed arteries. In genomic and metabolomic studies, the respiratory chain and mitochondrial pathways which generate ATP were leading pathways associated with AD. This review explores the notion that ATP depletion resulting from hippocampal hypoperfusion has a prime role in initiating damage. Sections cover sensing of ATP depletion and protective responses, vulnerable processes with very heavy ATP consumption (the malate shuttle, the glutamate/glutamine/GABA (γ-aminobutyric acid) cycle, and axonal transport), phospholipid disturbances and peroxidation by reactive oxygen species, hippocampal perfusion and the effects of hypertension, chronic hypoxia, and arterial vasospasm, and an overview of recent relevant genomic studies. The findings demonstrate strong scientific arguments for the proposal with increasing supportive evidence. These lines of enquiry should be pursued.

## 1. Introduction

**Figure 1:**
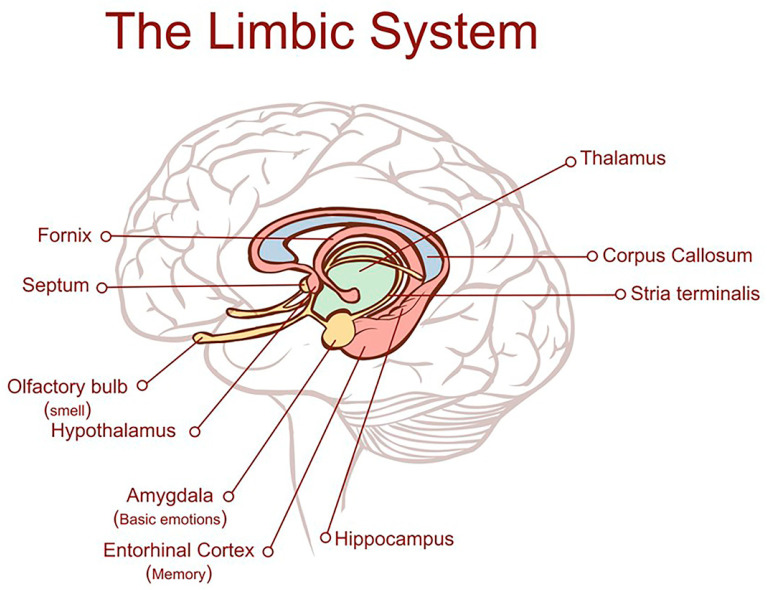
Location of the entorhinal cortex. The entorhinal cortex is part of the brain’s limbic system which controls emotional drives and memory formation. It is a collection of structures located deep within the brain which includes the hippocampal formation, amygdala, septal nuclei, cingulate cortex, entorhinal cortex, perirhinal cortex, and parahippocampal cortex. The last three cortical areas comprise different portions of the temporal lobe [6]; image ID HYTTNN hakan çorbac?/Alamy Stock Vector reproduced under license from Alamy Limited, Abingdon, UK, https://www.alamy.com. (accessed on 25 July 2025).

There is a common misconception that Alzheimer’s disease (AD) is merely an exaggeration of the changes which occur normally as the brain ages [1]. This is not the case. It is a distinct neurodegenerative disorder which arises within an epicentre in the medial temporal lobe of the brain and leads to characteristic protein accumulations, tau tangles, and amyloid plaques composed of conglomerations of toxic Aβ fragments of amyloid precursor protein, inflammation, destruction of synapses, and neuronal loss [2]. These abnormalities are superimposed on changes which occur normally with old age. The earliest changes observed, tau tangles and neuronal loss [1,3], are identifiable in later middle life in the entorhinal cortex (EC), a small region situated deeply in the medial temporal lobe adjoining the hippocampus indicated in Figure 1, which is the main interface between the hippocampus and the brain neocortex [4,5,6].

The EC/hippocampal system is responsible for creating declarative memories that can be consciously thought of, semantic memory (the ability to recall general facts about the world), memory formation and consolidation, and memory optimization in sleep [3,4,7]. From the EC, the damage extends slowly but relentlessly into the hippocampus, the cingulate gyrus, and the brain neocortex, eventually reaching the frontal cortical areas. By this time, there is considerable loss of both grey and white brain matter [8]. When tau tangles are first detectable in the EC, affected individuals may not have had memory problems or may have only minimal loss of memory and/or cognition (classed as MCI) [9]. However, compared with cognitively normal controls, AD-affected individuals with the mildest clinical dementia had 32% fewer neurons in the EC and 60% and 40% fewer in EC layers II and IV, respectively. In those with severe dementia, neurons in layers II and IV were approximately 90% and 70% fewer than controls. In contrast, cognitively normal subjects had no EC neuronal loss between the sixth and ninth decades of age [1]. Such a dramatic reduction in neurons must have started well before the onset of symptoms [1].

AD is a multifactorial disorder. Table 1 lists some of the manymany factors which have been shown to increase the risk for AD.

Although there is a genetic component to risk, only 1.5% to 2% of individuals with inherited defects in processing the amyloid precursor protein, APP, have a clear monogenic cause. They present at a young age and are not typical of the 98% of individuals with a later onset of AD (LOAD) [26]. It is apparent from the table that a high percentage of the world population has these risk factors and yet does not develop the disease [10,11]. To add further confusion, up to one-third of community-dwelling older adults have Tau tangles and/or amyloid deposits at autopsy but have experienced negligible cognitive decline in life [2]. Why does AD affect only some of those at risk? Why, of all brain regions, is the hippocampus/EC targeted in AD? Why does this happen in only some individuals? What triggers the process? What is happening during the asymptomatic prodromal phase? These are recurring questions being intensively investigated. Currently, the prevailing view (the amyloid cascade hypothesis) is that pathological accumulation of Aβ peptides is the causative event. This triggers downstream pathways leading to Tau misfolding and tangle formation and ensuing neurodegeneration, vascular damage, and loss of cognition [27,28]. Striking differences in the composition of the brain from other organs increase its vulnerability to pathogenic insult. First, it is wholly reliant on a continuous supply of ATP to function and has a relatively enormous energy requirement for its size, accounting for up to 20% of total body energy produced [9]. Hence it is totally dependent on mitochondria which can adapt instantaneously to changing needs. Second, it is a fatty organ. Ten per cent to 20% of the fresh weight and more than 50% of the dry weight is composed of lipids [Yin]. Of these, approximately 50% are phospholipids [29], which are the major lipid constituents of membranes [30]. Third it has an enormous area of membranes covering the cell surface and internal organelles. Hence the brain is particularly vulnerable to disturbance of lipid turnover; fourth, polyunsaturated fatty acids (PUFAs) are enriched in brain membrane lipids and have an essential structural role. However, they also provide an abundant substrate for cascading free radical attack causing peroxidative membrane damage [31,32]; fifth, it is the most cholesterol-rich body organ. Although accounting for only 2% of body weight, it contains around 25% of body cholesterol [33,34]. Most is synthesized in the brain and incorporated into membranes [35].

Decades of research studies have shown that many interlinked cellular and molecular disturbances contribute to the AD phenotype, notably neuroinflammation [11], mitochondrial dysfunction [16] and circulatory disturbances [36]. Incredible developments in gene and metabolite analyses, neuroimaging, and information technology have enabled deep probing of events in this inaccessible organ. Knowledge is advancing dramatically. It is now widely held that mitochondrial dysfunction has a major role in pathogenesis, and there is growing evidence that it is an impaired blood supply that targets initial damage to the hippocampus.

The review explores the notion that recurrent minor ischaemic events due to a reduction in hippocampal blood flow (hypoperfusion) cause episodes of ATP depletion. These are proposed to initiate cellular disturbances which cascade, provoke an inflammatory response, and result in cumulative damage. This combines two concepts which are not new, but it seems timely to review them in light of recent findings. There are four main sections: (i) a brief summary of changes which occur normally in an ageing brain; (ii) a large section covering sensing of ATP depletion, processes with very heavy ATP consumption (the malate shuttle, the glutamate/glutamine/GABA (γ-aminobutyric acid) cycle, and axonal transport), and the consequences of ATP deficiency on lipid turnover, membrane function, neurotransmission, and synapses; (iii) a section covering hippocampal perfusion, responses of cerebral blood flow to hypoxia, neuronal activity and intraluminal pressure, and reduced flow in hypertension and chronic hypoxia; and (iv) an overview of recent genomic studies relevant to the proposal. From the review, the conclusions are that there are strong scientific arguments for the proposal, that there is supportive evidence, and that these lines of enquiry should be pursued. Studies are already indicating new approaches for preventative interventions, early diagnosis, and therapy.

## 2. Brain Ageing

Brain ageing is characterized by a progressive loss of grey and white matter volume, a general loss of dendritic spines, loss of synaptic plasticity, increased rates of axonal bouton turnover, and inflammation [37]. However, the number of neurons is largely preserved in the neocortex and hippocampus of the ageing human brain [1,38].

Brain energy metabolism declines with age, affecting most brain regions [39]. Functional brain mitochondrial deficits that occur with age include decreased rates of respiration and of electron transfer, a continuous decrease in the capacity to produce ATP by oxidative phosphorylation [40,41], dynamic changes in shape and size, activation of the permeability transition pore [42], and loss of membrane potential [39]. Increased mitochondrial fusion with elongation was observed with age [43]. Mitochondrial fission factors were reduced in ageing mice [44] but were increased in synaptic mitochondria [44]. Expression of oxidative phosphorylation (OXPHOS) proteins in whole brain tissue decreases in advanced age, particularly of complexes I, IV, and V [39,40], but inner membrane H^+^ impermeability and F1-ATP synthase activity are only slightly affected [40]. Expression of TCA cycle proteins was lower in the whole brains of elderly mice [45] and rats [46] compared with middle-aged rodents. Pathway analysis of expressed genes identified the synaptic vesicle cycle, the GMP-PKG (cGMP-protein kinase G) signalling pathway, and oxidative phosphorylation as core gene sets showing the highest association with human brain ageing [47]. Production of mitochondrial superoxide, oxidative damage, and peroxidation of membrane lipids increases with age [48,49]. Significant changes in the expression of genes affecting synaptic function were observed in the human brain over the age range of 20–99 years, often showing progressive down-regulation [50]. With a highly sensitive imaging procedure, compared with young animals, glutamate levels were significantly lower in elderly lemurs in the hypothalamus and other brain regions, particularly the globus pallidus and nucleus accumbens [51].

## 3. A Role for ATP Depletion in the Genesis of AD

### 3.1. Matching ATP Production to Requirement in the Brain

The brain has a continuous high requirement for ATP which oscillates with neuronal activity and cellular stresses. The ATP supply must be adjusted constantly according to need [52]. This demands an efficient production system with a generous reserve capacity [53] which can respond immediately to instructions from a wide range of sources. In the brain, mitochondria are the main source of ATP which under normal circumstances is generated predominantly from glucose via pyruvate oxidized aerobically, with a smaller proportion from cytoplasmic glycolysis. The production unit comprises the five large complexes of the oxidative phosphorylation (OXPHOS) system tightly configured at the inner mitochondrial membrane. Construction of the system is a highly co-ordinated process. Between 1000 and 2000 mitochondrial-related genes have been identified in nuclear DNA [54]. Only thirteen of the OXPHOS peptides are encoded by mitochondrial genes. A mitochondrion contains 2–10 copies of mtDNA. The copy number (mtDNA-CN) varies within a cell and correlates with its tissue’s bioenergetic needs. The abundance of mitochondrial transcripts is higher in tissues with high energy demands [55]. Each somatic cell can have up to 1000 mitochondria. Studies have demonstrated a significant association of mtDNA-CN with TFAM (Mitochondrial Transcription Factor A). Other proteins that regulate copy number are TWINKLE, a mitochondrial DNA helicase, and POLG-A, a subunit of DNA polymerase γ. mtDNA is vulnerable to mitochondrial stressors, including oxidative stress, which can disturb the respiratory chain complexes, lead to the release of damaging reactive oxygen species (ROS), and reduce ATP production [54]. The tricarboxylic acid (TCA) cycle in the mitochondrial matrix supplies most of the fuel for oxidation as NADH^+^, but this is supplemented by many other biochemical processes, notably fatty acid oxidation which generates FADH_2_ [56].

### 3.2. Biosensors of ATP Status

#### 3.2.1. AMPK

AMPK is a serine/threonine kinase. It is the primary sensor for ATP status in the body through direct interaction with the adenine nucleotides AMP, ADP, and ATP [52]. AMPK is heterotrimeric with subunits α, β, and γ. The α subunit carries the catalytic domain; β and γ are regulatory [52]. A conserved Thr 172 in the activation loop of the kinase domain is regulated by at least three upstream kinases: LKB1 (liver kinase B1), CaMKK2 (calmodulin-dependent protein kinase kinase β), and TAK1 (TGFβ-activated kinase 1), and dephosphorylated by three phosphatases, PP2A (protein phosphatase 2A), PP2C (protein phosphatase 2C), and PPM1E (Mg^2+^/Mn^2+^-dependent protein phosphatase 1E) [52,57]. The γ-subunit has 4 tandem repeat motifs (termed CBS1 to CBS4), which assemble to form binding sites for AMP, ADP, and ATP. CBS3 seems to be the critical site. In an energy-replete state (low AMP/ATP and ADP/ATP ratios), phosphatases can access T172 and keep AMPK unphosphorylated and inactive, but phosphatase access is blocked when high levels of AMP or ADP bind to CBS3 in the γ subunit [58].

AMPK maintains and restores ATP when energy levels are falling. It is activated by increasing AMP or ADP coupled with falling ATP. The AMP/ATP ratio is affected by even small changes in AMP [58]. AMPK switches on numerous genes in catabolic pathways which generate ATP and switches off many in anabolic pathways which use ATP. Up-regulated genes include genes involved in mitochondrial fission, autophagy, mitophagy, mitochondrial biogenesis, glycolysis, glucose uptake, fatty acid uptake, fatty acid catabolism, branched-chain amino acid catabolism, and redox regulation, all relevant to this review. Relevant down-regulated genes include genes involved in RNA synthesis, protein synthesis and elongation, and the synthesis of triacylglycerol phospholipids, cholesterol, fatty acids, and glycogen [52,57,58,59]. AMPK also has a role in the metabolic responses to caloric restriction and exercise [52]. It is dysregulated in major chronic diseases including obesity, inflammation, and diabetes [58,59].

#### 3.2.2. Sirtuins

Sirtuins (SIRT) are a class of seven NAD^+^-dependent histone deacetylases that regulate gene transcription in many metabolic pathways [60,61,62,63,64]. Some also remove other acyl groups such as succinyl, malonyl, and long-chain fatty acyl groups [65]. Sirtuins differ in length and sequence in their C- and N-terminal domains. SIRT1 and SIRT2 localize in the nucleus and cytoplasm, and SIRT3, SIRT4, and SIRT5 are mitochondrial [62]. SIRT1 activity is regulated directly by cellular NAD^+^ levels and indirectly by AMPK activation which increases the intracellular NAD^+^/NADH ratio [66]. In turn, SIRT1 promotes AMPK activity by deacetylating and thereby activating LKB1 kinase and hence phosphorylation of AMPK [67]. A decrease in the NAD^+^/NADH ratio when glucose intake is high inhibits AMPK activation [58].

SIRT1 regulates a range of age-related processes including cellular senescence, DNA damage repair, mitochondrial function, and inflammation through histone deacetylation of inflammatory cytokines including NF-kB, HIF1a, AP-1, and P38MAPK [68]. NF-κB associates with accumulating Aβ peptides and activates an inflammatory response via acetylation of its p65 subunit. Deacetylation of p65 by SIRT1 may limit Aβ-provoked damage. SIRT1/AMPK activity was shown to play a key role in autophagy by inducing mitochondrial fragmentation, which slows the progression of neurodegeneration [60,69]. Raising the activity of the AMPK-SIRT1-PGC-1α pathway increases mitochondrial biogenesis. SIRT1 depletion accelerates the ageing process and increases susceptibility to age-associated diseases [62]. One action of the polyphenol resveratrol, which is present in grape skin and red wine, is to activate SIRT1 with neuroprotective effects [70]. SIRT3 regulates enzymes involved in fatty acid oxidation, activates the respiratory succinate dehydrogenase complex flavoprotein subunit A (SDHA), a component of complex II in the respiratory chain [71], and increases the activity of the mitochondrial enzyme superoxide dismutase 2 [72].

#### 3.2.3. Phosphofructokinase (PFK)

PFK, the first irreversible step in glucose degradation by glycolysis, produces fructose bisphosphate (FBP) which is then committed to pyruvate oxidation. FBP is a good indicator of glucose and energy availability [58].

#### 3.2.4. ATP Regulation by Mitochondrial Nucleotide Transporters

Adenine nucleotide translocase (ANT) is a large mitochondrial solute carrier family of proteins expressed in the inner mitochondrial membrane. In a 1:1 exchange, it imports ADP^3−^ into the mitochondrial matrix for conversion to ATP by ATP synthase and exports ATP^4−^ from the matrix to the intermembrane space. It does not alter the mitochondrial adenine nucleotide content. ANT undertakes equimolar exchange of vast amounts of nucleotides daily and is essential for life [73,74,75,76]. Humans express five ANT genes. ANT1 (SLC25A4) is the most abundant form in the brain and in other tissues with high oxidative activity such as heart and skeletal muscle [73,74,75,76]. ANT is incorporated with F_1_F_0_-ATP synthase and the phosphate carrier (PiC) proteins in the mitochondrial supercomplex (the ‘ATP synthasome’). It is also associated with respiratory chain supercomplexes [74]. AMPK has been implicated in the regulation of ANT activity via its interaction with SIRT4 [77] and via the ANT-AMPK-mTORC1 signalling pathway [78]. In addition to roles in energy provision, there is evidence that ANTs have more extensive roles in mitochondrial biology including regulation of the opening of the mitochondrial permeability transition pore (MPTP) and mitophagy [74].

ATP-mitochondrial ATP-Mg/Pi carriers are integral proteins of inner mitochondrial membranes which mediate electroneutral exchange of phosphate for adenine nucleotides coupled to magnesium or protons [73,79,80,81]. Their activity enables mitochondria to replenish adenine nucleotide pools depleted by cellular activities [79,81]. They are probably the only transporters responsible for net changes in mitochondrial adenine nucleotide levels. Humans have four carriers. SLC25A23 (Solute Carrier Family 25 Member 23), SLC25A24, and SLC25A25 are calcium-regulated [81,82]. Their N-terminals face the mitochondrial intermembrane space and hence can transduce cytoplasmic Ca^2+^ signals. The fourth carrier, SLC25A41, is not regulated by Ca^2+^ [66]. Fibroblasts from *slc25a25*-deficient mouse embryos had decreased mitochondrial ATP, basal mitochondrial respiration, and decreased Ca^2+^ flux across the sarcoplasmic reticulum [83]. Glucagon, vasopressin, epinephrine, and a low insulin/glucagon ratio transiently increase cytoplasmic Ca^2+^ by activating their receptors and increase glycolysis and glycogenolysis-derived ATP production and the ATP/ADP ratio. AMPK can influence mitochondrial ATP transporter activity indirectly through activation of membrane Ca^2+^ transporters. In renal tubular cells, SLC25A25 was shown to be activated by Ca^2+^ entry via the transient receptor potential cation channel PKD2 (polycystin 2) which is activated by AMPK [84,85]. In this study, knockdown of *SLC25A25* decreased cellular respiration and significantly reduced ATP concentrations, but had no effect on cell growth or survival. Compared to wild-type cells, there were significant changes in lipids, purine and pyrimidine nucleosides and nucleotides, and amino acids, notably with a large decrease in aspartate, and in intermediates of glutathione metabolism [85]. This provided a unique view of the effects of isolated ATP deficiency.

### 3.3. Hypoxia-Inducible Factor 1 (HIF-1) Mediates the Response to Hypoxia

The transcriptional complex HIF-1 plays an essential role in cellular and systemic oxygen homeostasis [86,87,88]. It induces the transcription of more than 60 neuroprotective proteins which promote erythropoiesis and angiogenesis, thereby increasing oxygen availability, glucose transport, and metabolism [86,89,90]. HIF-1 consists of a constitutively expressed HIF-1β subunit (Aryl Hydrocarbon Receptor Nuclear Translocator, ARNT1, HIF1ß) and one of three subunits (HIF-1α, HIF-2α, or HIF-3α). Under normoxic conditions, HIF-1α protein is degraded rapidly via the von Hippel–Lindau tumour suppressor gene product (pVHL)-ubiquitin-proteasome pathway [86]. The association of HIF-1α with pVHL is triggered by post-translational hydroxylation of proline residues mediated by prolyl hydroxylase (PHD) or HIF prolyl hydroxylase (HPH). PHD is a dioxygenase. Its activity depends on oxygen concentration and hence PHD has been proposed as the HIF-1α oxygen sensor. In hypoxic conditions, HIF-1α is stabilized, heterodimeric HIF-1α/β translocates into the nucleus and interacts with E1A binding protein p300/CREB-binding protein (p300/CBP) and other coactivators [91,92,93,94] which synergistically enhance HIF-1α transcription of target genes. Growth factors induce HIF-1α protein translation via PI3K (phosphoinositide 3-kinase) or MAPK (mitogen-activated protein kinase) pathways irrespective of hypoxia.

Genome-wide chromatin immunoprecipitation (ChIP) identified HIF 1-dependent increased or decreased expression levels of hundreds of genes in response to hypoxia [95]. Vascular endothelial cell growth factor (VEGF) and erythropoietin are major HIF1 target genes. Amongst many other transcriptionally activated genes are genes encoding cyclin, IGF2 (Insulin-like Growth Factor 2), IGFBP1 and IGFBP2 (insulin-like growth factor-binding protein 1 and 2), NOS2 (Nitric Oxide Synthase 2), GLUT1 and GLUT3 (Glucose Transporter 1 and 3), transferrin, the transferrin receptor [86] and caeruloplasmin [96]. VEGF specifically recruits endothelial cells into hypoxic and avascular areas and stimulates their proliferation. It is the most potent endothelial-specific mitogen and is known to directly participate in angiogenesis. HIF-1α has also been shown to indirectly contribute to Tau phosphorylation. Because up-regulated HIF-1α in chronic hypoxia decreased the activity of protein phosphatase-2A (PP2A), it was proposed that this may mediate Tau hyperphosphorylation with increased risk of resultant cognitive dysfunction [97].

#### Hypoxia Up-Regulated Mitochondrial Movement Regulator (HUMMR)

HUMMR is expressed in neurons and is markedly induced by HIF-1α [98]. It interacts with Miro-1 and Miro-2, mitochondrial proteins that are critical for mediating mitochondrial transport (refer to Section 4.3). Knockdown of HUMMR or HIF-1 function in neurons exposed to hypoxia markedly reduced the mitochondrial content in axons. The percentage of motile mitochondria moving in the anterograde direction decreased and the percentage moving in the retrograde direction was enhanced [98].

### 3.4. Mitochondrial-Derived Peptides (MDPs) and Nuclear-Encoded Microproteins

The discovery of small bioactive signalling peptides coded by mitochondrial DNA (MDPs) over the last 15 years has radically changed our vision of the roles of mitochondria in directing cell metabolism [17,99,100]. MDPs are encoded by short open reading frames (ORFs) from noncanonical transcription sites within the known mitochondrial genes [55]. So far eleven have been reported: humanin and six small humanin-like peptides with 20–35 amino acids (*SHLP1–SHLP6*) are encoded within *MT-RNA2,* the gene for 16S rRNA [101]. *The mitochondrial open reading frame of the 12S rRNA-c (MOTS-c)* was identified within the *12S rRNA* gene and codes for a 16 amino acid peptide [102]. Other MDPs are *SHMOOSE* (*Small Human Mitochondrial ORF Over SErine tRNA)* and *GAU* (*gene antisense ubiquitous)*. In addition, a 99 amino acid polypeptide is translated from an alternative reading frame in the mtDNA gene *MT-CYTBmt* [103]. In vitro humanin is antiapoptotic and increases mitochondrial respiration, cell proliferation, and cell survival through cell membrane receptors [99,104]. SHLP2 and SHLP3 also decrease apoptosis. SHLP2 has a protective interaction with mitochondrial complex 1 and increases mitochondrial respiration and ATP levels [17,103]. MOTSc, however, decreases mitochondrial respiration and increases glycolysis in vitro [103], and MtALTND4, encoded from an alternative open reading frame of the gene for *ND4*, a subunit of NADH dehydrogenase (Complex 1), decreased the O_2_ consumption rate, maximum coupled and uncoupled respiration, and spare reserve capacity of Hela and HEK-293 cells [103].

Microproteins encoded within nuclear genes which modify mitochondrial function are also being recognized. A uORF located in the 5’ UTR of the nuclear gene for mitochondrial dynamics protein 1 (*MID51, Mitochondrial Elongation Factor 1 (MIEF1*)) encodes a mitochondrial microprotein [70 amino acids], named MIEF1-MP (MIEF1-microprotein) that is involved in mitochondrial fission and interacts with the mitochondrial ribosome [105]. Independently, three groups identified a microprotein (56 aa) encoded by a lncRNA (gene *MTLN*) which they named mitoregulin [106], MOX1 [107], and MPM [108]. Mitoregulin supported super complexes and modified mitochondrial respiratory efficiency [99,106]. Mitochondrial- and nuclear-encoded micropeptides/microproteins are likely to have important physiological roles in regulating cellular stress responses, apoptosis, and metabolic processes and to be under epigenetic control.

A polymorphism of the humanin gene with an incidence of 1–5% in individuals with African ancestry was shown to associate with lower plasma humanin levels and greater cognitive decline [17,109] Surprisingly, this SNP is common among individuals of European descent, with an incidence approaching 50%, and so far has not been associated with dementia, which must reflect the multifactorial nature of the condition. A second humanin variant found in individuals of Ashkenazi descent promoted higher affinity binding to APOE4 than the more common allele in vitro, and in mice expressing human APOE4, the variant reduced AD-related pathology more effectively [110].

Table 2 summarizes important actions of AMPK and of other ATP sensors.

### 3.5. Spectrum of Molecules Involved in ATP Turnover

Bennett, Nguyen, and Darch et al. [112,113] developed a fluorescence-activated cell sorting (FACS)-based assay to screen the genome for regulators of cellular ATP levels of K562 leukaemic cells expressing a fluorescent ATP biosensor. They screened the entire genome with CRISPR interference and CRISPR activation libraries and isolated cells with high and low ATP levels under basal conditions or cells dependent on mitochondrial ATP generation (glycolysis inhibited) or on glycolytic production (respiration blocked). They identified numerous gene pathways and ontologies that impacted ATP when knocked down or over-expressed. One of relevance was *HSD17B10* (Aβ-binding alcohol dehydrogenase, ABAD) which is involved in isoleucine and neurosteroid metabolism, is up-regulated in Alzheimer’s disease, and has been shown to interact with Aβ peptide but it is unclear whether they have concerted or independent roles in AD pathogenesis [114]. Of considerable interest was their demonstration that HIF1α and aryl hydrocarbon nuclear translocator (ARNT1, HIF1ß), which form the functional HIF1 molecule, were at the centre of a network which included the HIF1 targets HK2 (hexokinase 2) and binding partner VDAC1 (voltage-dependent anion channel 1) [115], and the HIF1-regulating proteins SENP1 (SUMO-specific peptidase 1) and SP1(Sp1 transcription factor) and upstream genes that regulate HIF1.

There is increasing interest in extracellular ATP which may be released into the extracellular space in response to cell stimulation and act as a signalling molecule. ATP activates P2XR purine receptors which are expressed in neurons and glial cells, and promotes Ca^2+^ entry and neurotransmitter release. It has been proposed that ATP may also leak from cells damaged by inflammation associated with Aβ peptide, and that this might precipitate neuronal hyperstimulation [111]. An alternative fate for extracellular ATP is enzymatic catabolism into adenosine monophosphate (AMP) which is then cleaved to adenosine by ecto 5-nucleotidases. Adenosine binds to adenosine A1 receptors (A1Rs). Post-synaptic binding inhibits the activation of glutamatergic *N*-methyl-d-aspartate receptors (NMDARs) and is neuroprotective. Increased HIF-1 production in hypoxic conditions promotes this process by activating ecto 5-nucleotidases, and thereby prevents hyperstimulation and excessive ATP consumption [111].

## 4. Brain Processes with Very High ATP Consumption/Turnover

There are three intimately linked pathways in the brain with very high ATP usage demanding continuous replenishment: the malate-aspartate shuttle, the glutamate/glutamine cycle, and axonal transport/synaptic transmission. These are at high risk of disruption by oxygen depletion with severe consequences for brain function.

### 4.1. The Malate-Aspartate Shuttle

**Figure 2:**
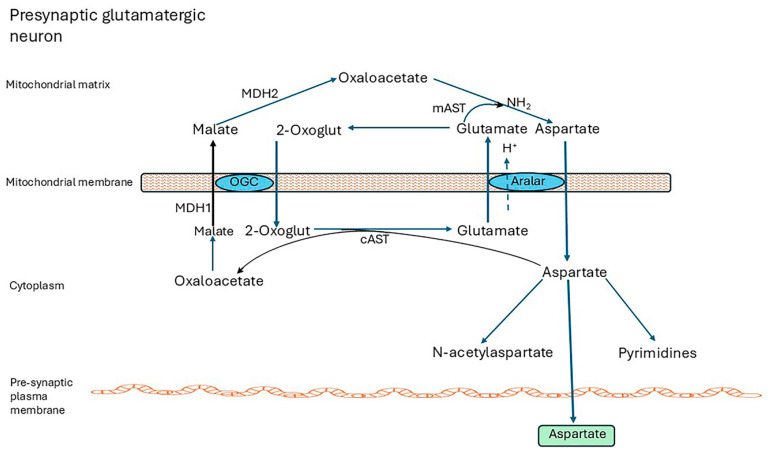
The malate-aspartate shuttle. NADH cannot be transported from the cytosol into mitochondria. To regenerate NAD from NADH produced during oxidative reactions in the cytosol, H^+^ from NADH is transported into mitochondria via the malate-aspartate shuttle (MAS). MAS requires two cytoplasmic enzymes, cAST and MDH1; two mitochondrial enzymes, mAST and mMDH2; and two carriers located in the inner mitochondrial membrane, the aspartate-glutamate carrier aralar (SCL25A12) and the 2-oxoglutarate carrier OGC (SLC25A11). (i) In the cytoplasm, MDH1 transfers reducing equivalents from NADH to oxaloacetate, producing malate; (ii) OGC transports malate into mitochondrion in exchange for 2-oxoglutarate (2-oxoglut); (iii) mMDH2 then oxidizes malate to oxaloacetate (OAA), generating NADH; (iv) mAST transfers NH_2_ from glutamate to OAA producing aspartate and 2-oxoglut; (v) aralar transports aspartate out into the cytoplasm in exchange for glutamate and H^+^ into the mitochondria; and (vi) finally, cAST transaminates 2-oxoglut forming OAA and glutamate, closing the cycle. After entry into mitochondria, electrons are supplied to the electron transport chain in the form of NADH for ATP production, and cytosolic NAD^+^ is regenerated [116,117,118]. The Glutamate/H^+^ symporter, SLCA22, may also contribute to the shuttle activity [18]. Abbreviations: MDH1 malate dehydrogenase 1, MDH2 malate dehydrogenase 2 (mitochondrial), mAST mitochondrial aspartate aminotransferase, alias GOT2 glutamic-oxaloacetic transaminase 2, mitochondrial, cAST cytoplasmic aspartate aminotransferase, alias GOT1 glutamic-oxaloacetic transaminase 1, 2-Oxoglut, 2-oxoglutarate, OGC 2-oxoglutarate carrier.

Intact mitochondria are impermeable to NADH; hence reducing equivalents generated as NADH in the cytosol by NAD^+^-dependent pathways must enter the mitochondria indirectly. In the brain this is via the malate-aspartate shuttle (MAS) which operates in neurons but not in astrocytes. The other major redox shuttle (glycerol-3-phosphate shuttle) has very low activity in the brain. Figure 2 shows the shuttle in a presynaptic glutamatergic neuron. The legend explains how it operates.

Astrocytes do not express aralar and lack a complete shuttle. The closely related aspartate-glutamate carrier, AGC2 (SLC25A13, citrin), associated with the urea cycle in the liver [118], is not expressed in the brain. Aralar is regulated by cytosolic Ca^2+^, and small cytosolic Ca^2+^ signals activate the Aralar/MAS pathway [119,120].

The MAS is essential for maintaining redox balance in the cytosol and mitochondria, for securing and transferring the energy generated as NADH in the cytosol, and for neuronal use of lactate as fuel. By regenerating NAD^+^, it enables the activities of cytosolic enzymes to continue and other NAD^+^ functions such as signalling and regulation of transcription by sirtuins [116]. Importantly, it generates aspartate for export from neurons for subsequent uptake by astrocytes where it has a central role in glutamine synthesis (refer to Section 4.2). Aspartate is also essential for pyrimidine synthesis [121] and is converted to N-acetylaspartate (NAA) and exported to oligodendrocytes, where it is de-acetylated and metabolized, and provides acetate for fatty acid synthesis [117,122,123]. In humans, aralar deficiency presents with severe infantile-onset encephalopathy with epilepsy, global developmental delay, generalized hypotonia, loss of cerebral volume, diffuse brain atrophy, hypomyelination/white matter loss, and reduced cerebral NAA on brain imaging [25,117,124,125]. Infants lacking other shuttle enzymes, GOT2, MDH1, or MDH2, have exhibited similar symptoms [116,126,127,128], which are mirrored in *aralar-*KO mice [129,130]. Brain Asp levels of KO mice were 80% to 90% lower than controls. Asp and NAA levels of brain and cortical neuronal cell cultures from all brain regions of KO animals were drastically decreased and alanine (Ala) and serine (Ser) were severely reduced [131].

### 4.2. The Glutamate/GABA/Glutamine Cycle

**Figure 3:**
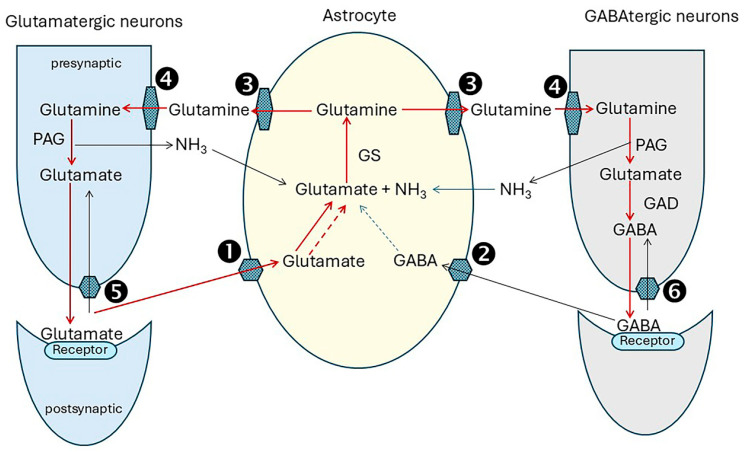
Overview of the glutamate/GABA/glutamine cycle. The GABA–glutamine–glutamate shuttles replenish the neurotransmitters, L-glutamate and GABA using glutamine generated in astrocytes. After release from glutamatergic neurons into the synaptic clefts, glutamate not bound to post-synaptic receptors is carried with Na^+^ and H^+^ into astrocytes by the glutamate–aspartate transporters SLC1A3 (solute carrier 1A3, EAAT1) and SLC1A2 (EAAT2), step ①. Similarly, unbound GABA released from GABAtergic neurons is carried into astrocytes with sodium and chloride by the GABA transporter SLC6A11 (GAT3), step ②, where it is converted to glutamate. Glutamine synthetase (GS) then catalyzes the formation of glutamine from glutamate and ammonia in an ATP-dependent reaction. Glutamine is exported with sodium into the extracellular space by Na^+^-amino acid cotransporters, SLC38A1 (SNAT1) and SLC38A2 (SNAT2), and Na^+^-amino acid cotransporters-H^+^ antiporters, SLC38A3 (SNAT3) and SLC38A5 (SNAT5), step ③, and then imported into neurons by one or more SNAT transporters: SNAT1, 2 and SNAT7 [SLC38A7]), step ④. In glutamatergic neurons glutamine is hydrolysed by PAG (phosphate-activated glutaminase). In GABAtergic neurons. glutamine is dehydrogenated by GDH (glutamate dehydrogenase), producing GABA. The neurotransmitters are then packaged into vesicles, transported to the synapses, and released with neuronal stimulation. Most neurotransmitters not bound to post-synaptic receptors are recycled via astrocytes as described above. A fraction is transferred back into neurons: glutamate by SLC1A3 ⑤ and GABA by SLC6A1 (GAT1) ⑥. Mitochondrial glutamate/H^+^ symporter SLCA22 probably makes a significant contribution to the cycle [18]. Abbreviations: Solute carrier family members (SLC): SLC1A3 aliases excitatory amino acid transporter 1 (EAAT1), glutamate-aspartate transporter (GLAST), SLC1A2, aliases EAAT2, glutamate transporter 1 (GLT1), SLC6A1 (alias GABA transporter 1 (GAT1), SLC6A11, alias GABA transporter 3 (GAT3); Na^+^-amino acid cotransporters: SLC38A1 (SNAT1) and SLC38A2 (SNAT2); Na^+^-amino acid cotransporters-H^+^ antiporters, SLC38A3 (SNAT3), SLC38A5 (SNAT5), and SLC38A7 (SNAT7). Red lines depict the major pathways, grey lines additional routes. Solid lines indicate direct enzyme conversions; dotted lines depict production from TCA cycle intermediates.

Glutamine (Gln) and GABA for neurotransmission are synthesized and replenished by the interaction of glutamatergic and GABAtergic neurons and astrocytes in a tightly co-ordinated sequence termed the glutamate/GABA/glutamine cycle. Because neurons lack the capacity to synthesize glutamate (Glu) de novo, astrocytes are the primary regulators of Glu and GABA biosynthesis [132,133]. Figure 3 shows the sequence of events. The legend explains how it operates and lists the main transporters and enzymes involved.

Unbound neurotransmitters must be cleared quickly from the synaptic cleft to prevent neurotoxicity from excessive stimulation. There are many members of the solute carrier (SLC) family, and there is current uncertainty about their relative contributions to the cycle processes, particularly in the transfer of glutamine from astrocytes to neurons [134]. Glutamine synthetase (GS) is expressed abundantly in the fine astrocytic processes associated with glutamatergic synapses in the rat hippocampus [135]. NH_3_ for glutamine synthesis is supplied by phosphate-activated glutaminase (PAG) activity and by other enzyme processes such as inosine monophosphate degradation in the purine nucleotide cycle which is active in the brain [136], and from the systemic circulation [136]. Glutamine-glutamate cycling accounts for approximately 80% of total neurotransmitter recycling, and glutamine-GABA cycling for the remainder [132].

**Figure 4:**
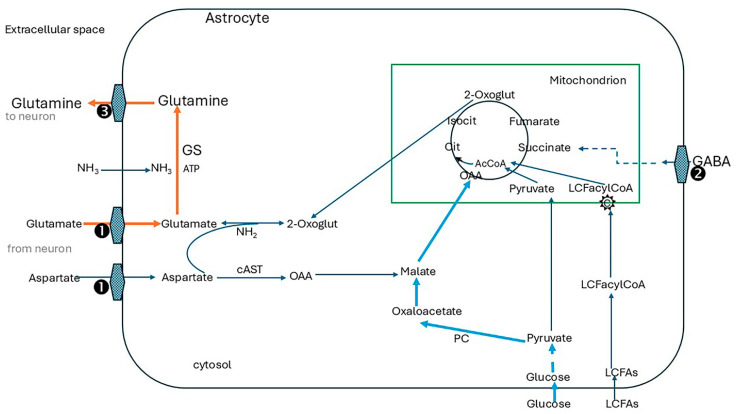
Astrocytes are central to neurotransmitter turnover of glutamatergic neurones. Glutamine is replenished in astrocytes by transamination of 2-oxoglutarate drawn from the TCA cycle, producing glutamate which GS then converts to glutamine. TCA cycle function is maintained largely by carboxylation of pyruvate (provided by glucose) by cytosolic pyruvate carboxylase forming oxaloacetate. This enters the cycle after hydrogenation to malate and transport into mitochondria. Oxaloacetate is also produced from aspartate generated by the malate-aspartate shuttle and makes an essential contribution to anaplerosis. Further anaplerotic support is provided by reuptake of GABA into astrocytes and its conversion to succinate. ① SLC1A3, alias excitatory amino acid transporter 1 (EAAT1; GLAST), SLC1A2, alias excitatory amino acid transporter 2 (EAAT2, GLT1), ② SLC6A11 (GAT3), ③ Na^+^-amino acid cotransporters, SLC38A1 (SNAT1) and SLC38A2 (SNAT2), and Na^+^-amino acid cotransporters-H^+^ antiporters, SLC38A3 (SNAT3) and SLC38A5 (SNAT5). cAST cytoplasmic aspartate aminotransferase (alias GOT1 glutamic-oxaloacetic transaminase 1, cytoplasmic), GS glutamine synthetase, PC pyruvate carboxylase, OAA oxaloacetate, 2-oxoglut 2-oxoglutarate, cit citrate, isocit isocitrate, AcCoA acetyl CoA, LCFA long-chain fatty acids, LCFacylCoA, and C carnitine shuttle. Red lines, main route for glutamine synthess; solid blue lines, main anaplerotic pathway from glucose via pyruvate carboxylase; blue dotted lines anaplerosis from GABA via conversion to succinate.

By releasing glutamate and GABA, neurons are at the frontline of neurotransmission. However, their capacity to do this is entirely dependent on support from adjoining astrocytes to maintain the glutamine flow [133,137]. This involves complex biochemical interactions. The problem is that the glutamate/GABA/glutamine cycle is a leaky system. Not all of the glutamate from glutamine transferred into neurons is used for neurotransmission. Some 10% to 30% is diverted to cell metabolism [138]. Without replacing this, the neurotransmitter pool would be exhausted very rapidly [132]. Astrocytes bolster supplies by withdrawing 2-oxoglutarate (2OG) from their TCA cycle. Transamination by cytosolic AST yields glutamate for glutamine synthesis. However, this depletes the TCA of an essential component with the risk of rapid malfunction. This is prevented by anaplerotic activities [139] shown in Figure 4 and described in the legend. Pyruvate carboxylase, which carboxylates pyruvate to oxaloacetate, has a central role in anaplerosis. It is expressed abundantly in astrocytes [140,141].

Glucose oxidation supplies pyruvate for this pathway according to demand and is closely matched by transport of glucose into the cells, mainly by the glucose transporter GLUT1. Astrocyte function is further supported by glycogen which is a local energy reserve [142]. Aspartate generated by the malate-aspartate shuttle (Figure 2) is an essential contributor to anaplerosis. Further anaplerotic support is provided by the reuptake of GABA into astrocytes and its conversion to succinate [133] (refer to Figure 4).

#### 4.2.1. The Energy Cost of the Glutamate/GABA/Glutamine Cycle

The transport of glutamate into astrocytes has a high energy requirement which makes a significant demand on astrocytic metabolism. For each imported glutamate molecule, three Na^+^ ions move down their concentration gradient, accompanied by the inward movement of one H^+^ and the counter-transport of a K^+^ ion [143]. This results in significant depolarization of the astrocyte membrane [144,145]. The membrane is repolarized by K^+^ channels and by the Na^+^/K^+^ ATPase [146], which transfers 3 Na^+^ ions out of the cell and 2 K^+^ ions inward for each molecule of ATP hydrolysed [147]. In astrocytes, there is a close physical association between glutamate transporters, the Na^+^/K^+^ ATPase, and mitochondria [148,149].

#### 4.2.2. Disturbances of the Glutamate/GABA/Glutamine Cycle in AD

The activity of GS is vulnerable to mixed-function oxidation which rises exponentially with age [132]. The enzymatic activity of GS was reduced in brain samples of AD patients [150,151,152] and the APP/PS1 mouse model of AD [153,154]. In vivo, compared to controls, GS was found to be significantly oxidized in the hippocampus of individuals with MCI or AD [152]. In vitro, the Aβ peptide inhibits purified GS [155], as well as GS activity in cortical homogenates [156] and cultured astrocytes [157]. GS was one of the cellular proteins most prone to oxidation after Aβ_1-42_ treatment in vitro [158]. Expression of the glutamine transporters SNAT1 (sodium-coupled neutral amino acid transporter 1) and SNAT3 was decreased in the APP/PS1 mouse [153,154], and in vitro Aβ exposure leads to down-regulation of SNAT1 in cultured cortical neurons [159]. GS expression was reduced in the frontal cortex of 3xTG mice prior to significant Aβ accumulation (1 month of age) [160], indicating that GS dysfunction occurs early in AD development. Glutamine synthesis was reduced in hippocampal slices of 5xFAD mice at an early stage of the disease (2 months of age) [161]. With advanced disease at 8 months of age, glutamine synthesis was reduced in both hippocampal and cerebral cortical slices of 5xFAD mice [162]. Reduced glutamine/glutamate levels have been reported in the AD brain [158]. Expression of glutamate transporters, particularly *SLC1A2 (EAAT2)*, is severely reduced in the AD brain, resulting in reduced astrocyte glutamate uptake and potential excitotoxicity [163,164,165]. In three independent cohorts, the glutamate carrier *SLC25A22* was identified as a susceptibility gene for AD, and down-regulation was also associated with hippocampal atrophy. It was hypothesized that even a small decrease in *SLC25A22* could compromise neuronal and mitochondrial glutamate metabolism causing energy deficiency [18].

Hyperammonaemia has been proposed as a pathogenic factor in AD, but the mechanisms are unknown [14,15,166]. Increased NH_3_ levels could impact the glutamate/GABA/glutamine cycle by decreasing SNAT3 expression [167] or by increasing ATP consumption during detoxification by GS [136]. Increased serum and CSF levels of ammonia have been reported in AD patients. Asymptomatic women with mild chronic hyperammonaemia due to mutations of X-linked ornithine transcarbamylase (OTC), a urea cycle enzyme, show subtle changes on brain MRI [168]. However, hippocampal changes have not been reported, and neither has an association with AD to date. Mild cognitive impairment may be evident with formal testing [169,170].

#### 4.2.3. Effects of Hypoxia/Ischaemia on the Glutamate/GABA/Glutamine Cycle

Glutamate receptor antagonists have been shown to protect neurons from global and/or focal ischaemia, which is proposed to increase extracellular glutamate accumulation, leading to excessive activation of glutamate receptors and excitotoxic cell death [163]. Astrocytes are more resistant to hypoxia/ischaemia than neurons. Depending on their location relative to a focal ischaemic infarct, astrocytes undergo a progressive change in morphology, becoming ‘reactive astrocytes’ with loss of highly branched processes, hypertrophy, and increased expression of glial fibrillary acidic protein (GFAP) [146]. Transient oxygen/glucose deprivation caused relatively rapid fragmentation of mitochondria in the astrocyte processes, followed by a gradual decrease in number [171]. Decreased levels of *SLC1A2 (EEAT2)* and/or *SLC1A3 (EEAT1)* mRNA and/or protein were observed in models of hypoxia/ischaemia [172].

#### 4.2.4. Promoting Anaplerosis in Astrocytes to Support Glutamine Synthesis

Triheptanoin, an edible odd-chain fatty acid triglyceride, C7:0, can be used as a dietary supplement. The main metabolic product, heptanoate, crosses the blood–brain barrier (BBB) and enters mitochondria to increase succinyl-CoA abundance [173]. Heptanoate can also be converted to five-carbon ketone bodies or glucose via gluconeogenesis in the liver providing additional substrates for the TCA cycle in the brain [174]. Hence triheptanoin supports the TCA cycle through anaplerosis and by fuelling the cycle, hence potentially enhancing ATP production. Triheptanoin has been used in clinical trials for the treatment of neurological disorders including glucose transporter type 1 deficiency syndrome (GLUT1 DS) [175]. Treatment of 5×FAD mice with triheptanoin from 3.5 m of age for 4.5 m rescued brain ATP content, increased mitochondrial NADH abundance, respiration, and redox balance, and preserved synaptic density in the hippocampal CA1 region and entorhinal cortex, but did not decrease Aβ load or tau phosphorylation [176]. Triheptanoin administration combined with a high-protein ketogenic diet in APP/PS1 mice with AD-like pathology prevented cognitive deficits and astrogliosis [177].

### 4.3. Axonal Transport Has a High Energy Requirement

Organelles and proteins are generally assembled in the body of neurons and must be transported to synapses in the nerve terminals when required. Material from the synapses requiring neuronal processing must, similarly, be transported in the reverse direction. These functions are carried out by highly co-ordinated events initiated by cell signalling which are tightly regulated by posttranslational modifications of microtubules, and by organelle-specific interactions [178,179]. For each journey, the fundamental requirements are a track, a motor, and adaptors [180] to attach the cargo. There are two types of track. One is composed of actin filaments. These may be used for short-distance transport, as in dendrites, and are often associated with actin networks near the cell surface. The others are microtubules. They are composed of α- and β-tubulin molecules which dimerize and then polymerize into parallel protofilaments. These wrap around each other to form a microtubule with a ‘plus’ end orientated toward the distal axon and a ‘minus’ end toward the cell body. They are not permanent structures but assemble and disassemble according to need. Tau protein is an essential binding partner which regulates the bundling of the microtubules and stabilizes them [181]. Acetylation of microtubules by α-tubulin *N*-acetyltransferase (ATAT) may promote stabilization and additionally confer flexibility [182,183,184]. They are deacetylated by histone deacetylase 6 (HDAC6) and sirtuin-2 (SIRT2) [185].

The trafficking system is heavily used, transporting a wide variety of intracellular organelles, including endosomes, lysosomes, autophagosomes, secretory vesicles, mitochondria, proteins, and macromolecules. These attach to specific flexible adaptors which bind them to motors on the tracks for transport. Motor proteins are classified into three families: myosins, kinesins, and dyneins. The heavy chain of each motor type has a family-specific conserved head domain that binds to the filaments and generates force and motion through cycles of ATP hydrolysis. Organelle movement by myosins can be directed toward the actin filament plus ends, for example, by myosin V, or minus ends, for example, by myosin VI [186].

Kinesin motors generally mediate anterograde axonal transport and dynein drives retrograde axonal transport [178]. The Kinesin-1 family consists of three proteins. Of these, *KIF5A* is primarily expressed in neurones. It is composed of two heavy chains and two light chains. The heavy chain binds microtubules with the head domain and hydrolyses ATP near the N-terminus. The head is joined to a long divergent stalk with two coiled-coil domains, and a C-terminal tail associates with the cargo-binding light chains [179,187,188]. The stalk sequences facilitate homodimerization of the heavy chains that allow the motor to ‘walk’ by alternating cycles of heavy chain to filament binding, such that one head is always attached to the filament [189]. Each ‘step’ consumes ATP. Glycogen synthase kinase-3β (GSK3β) phosphorylates the KIF5A heavy chain to inhibit axonal transport and also phosphorylates the light chain to release cargoes [190,191]. The stress-activated protein kinases c-Jun N-terminal kinase 3 (JNK3) and p38 mitogen-activated protein kinase (p38 MAPK) were also shown to directly phosphorylate the heavy chains and to inhibit anterograde transport [192,193]. Cytoplasmic dynein (referred to as dynein) is a large, 1.4 MDa multimeric complex composed of dimerized heavy chains, two intermediate chains, two light intermediate chains, and additional light chains. The heavy chain binds to the light chains, to a linker connected to the motor, and to cargo via interaction with other dynein subunits at its N-terminal tail [187]. To activate the motor, dynein binds to dynactin, an adaptor complex [179].

#### 4.3.1. Axonal Transport of Mitochondria

**Figure 5:**
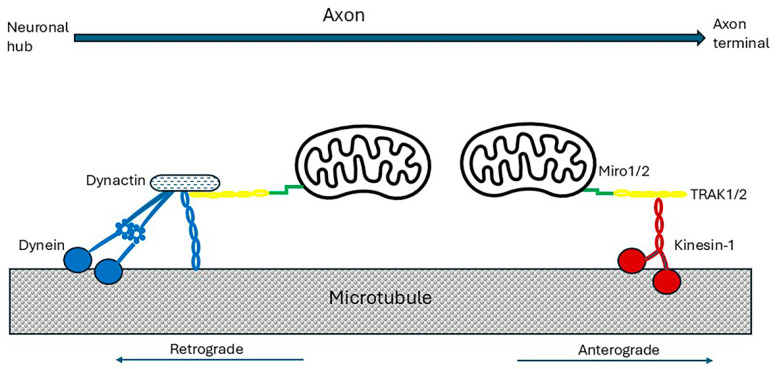
Axonal transport of mitochondria. For transport down axons, mitochondria attach to an adaptor which, in turn, links them to a motor bound to microtubules. Miro proteins (green) anchored to the outer mitochondrial membrane bind to trafficking kinesin protein adaptor complex adaptors, TRAK1 or TRAK 2 (yellow). For anterograde travel to the nerve terminals, TRAK1/2 binds to the kinesin-1 motor (red). This has two heavy and two light chains. The heavy chains dimerize and their head domains in alternation bind and then detach from myosin, consuming ATP, and ‘walk’ down the microtubules. Their C-terminals bind to the cargo-carrying light chains. The multimeric complex dynein and its activator dynactin (blue) transport damaged mitochondria from the axon terminals to the cell body for elimination by mitophagy [178,179,186,187].

Mitochondria in the neuronal cell bodies are transported down axons in response to changes in the local energy state and metabolic demand [178,186]. The transport mechanisms are the same as for other organelles. Increased neuronal Ca^2+^ released in response to neurotransmitter stimulation inhibited the motility of mitochondria without affecting the motion of other organelles [186]. Mitochondrial fusion/fission events and organelle size have an important influence on mitochondrial motility [186]. Clearly, there is close two-way communication between the neuronal cell body and its mitochondria, probably mediated via gene transcription [refer to Section 3.4]. Microtubule and myosin motors are bound to the mitochondrial surface by a conserved Miro-trafficking kinesin protein (TRAK) adaptor complex [194]. TRAK1 and TRAK2 bind directly to Miro proteins which are anchored to the outer mitochondrial membrane via a C-terminal transmembrane domain [195,196], as shown in Figure 5.

Mammals express two Miro (Mitochondrial Rho GTPase) proteins, Miro1 and Miro2. Both have two Ca^2+^-sensing EF-hand domains [195,197,198] and can act as Ca^2+^ sensors to induce Ca^2+^-dependent mitochondrial immobilization. Miro-1 and Miro-2 also interact with hypoxia up-regulated mitochondrial movement regulator (HUMMR), which is expressed in neurons and is markedly induced by hypoxia-inducible factor 1 α (HIF-1α) (refer to Section 3.3). In hypoxic conditions it facilitates anterograde, and represses retrograde, mitochondrial transport. Knockdown of HUMMR or HIF-1 in neurons exposed to hypoxia markedly reduced the mitochondrial content in axons [98,186]. Damaged mitochondria in the axon terminals are transported by dynein to the cell body/soma for elimination by mitophagy [199,200]. Defective retrograde transport of senescent mitochondria results in increased autophagy in axonal swellings [201].

#### 4.3.2. Role of Tau Protein in Axon Transport

Tau (microtubule-associated protein Tau) is encoded by the *MAPT* gene. Its normal physiological role is to induce tubulin assembly and stabilize microtubules, promote axonal growth, and enable axonal transport [202]. In vitro, it also binds to microtubules and actin simultaneously, promoting co-organization and coupled growth of both transport networks [203]. Tau is an intrinsically disordered protein consisting of an N-terminal, a proline-rich ‘projection’ domain, a microtubule-binding domain (MTBD) which incorporates three or four repeated motifs numbered R1 to R4, and a C-terminal tail. The motifs interact with the microtubules. Mutations within the MTBD impair tau-mediated microtubule stabilization [181]. Two of the motifs are conserved hexapeptides, named paired helical filament domains (PHFs). From fluorescence resonance energy transfer (FRET) studies, when not attached to microtubules soluble Tau molecules displayed an unfolded structure. When associated with microtubules, Tau monomers folded, decreasing the distance between the N and C termini [202]. This resulted in the formation of hairpin-like structures which stabilized a microtubule-bound conformation [204]. The findings strongly suggest that Tau’s capacity to regulate microtubule bundling and stabilizing activities is tightly controlled by its phosphorylation state [181]. Tau is thought to detach from microtubules through hyperphosphorylation of epitopes in the proline-rich domain and C-terminus of the Tau protein [205] by specific kinases such as glycogen synthase kinase-3β (GSK-3β), cyclin-dependent kinase 5 (CDK5), and phosphokinase A (PKA) [206,207]. AMPK has been linked to the control of mitochondrial anchoring at presynaptic boutons in mature cortical neurons, coupling local metabolic needs with mitochondrial positioning [207,208].

Tau detachment destabilizes microtubules and they disaggregate. Hyperphosphorylated tau is consistently associated with pathological lesions in human AD post-mortem material and in PET brain imaging, and with pathology and toxicity in animal studies [205,209]. Reactive microglia are often observed near NFTs which are thought to act as danger-associated molecular pattern (DAMP) molecules, further activating microglia and triggering an immune response. This could contribute to the chronic inflammation observed in AD [206]. However, p-Tau has been demonstrated in the human brains of individuals without AD, and its role in AD pathogenesis is unclear [205]. Tau has additional functions in axonal transport. It localizes in the cell nucleus, binds to histones, and may be involved in chromatin remodelling, or chromatin compaction, and is thought to protect DNA from damage. Misfolding or hyperphosphorylation of Tau would prevent this. Heat or oxidative stress causes nuclear translocation of Tau [210,211].

#### 4.3.3. Disordered Axonal Transport in AD

Dystrophic axons and axonal swellings, areas of expanded axons with accumulation of cargoes and motor proteins, are found in the early stages of AD in brains at autopsy and in an AD mouse model [179,212]. Mouse models with familial AD mutations show axonal pathology before Aβ plaque formation or NFT formation [179]. Dysregulation of axonal transport occurs early in neurodegenerative diseases and plays a key role in axonal degeneration [179]. Defective transport of axonal mitochondria is implicated in human neurological disorders and neurodegenerative diseases [212]. Loss of mitochondria from axonal terminals in Drosophila results in impaired synaptic transmission [178]. There is evidence that GSK3β kinase is hyperactive in AD [213]. Synapses are essential for transmitting, processing, and storing information, which all decline in ageing and AD [50]. Microarray analysis of brains collected at autopsy from non-AD controls aged 20 to 99 years and individuals with various psychiatric disorders, including AD, identified significant changes in the expression of numerous synapse-related genes, with many progressively down-regulated across ageing [214]. The widespread changes in synaptic gene expression in normal ageing suggested that the function of synapses might be impaired, and that ageing and AD share a common set of vulnerable synaptic genes [50].

## 5. Effects of ATP Depletion on Lipid Metabolism

The brain has an exceptionally high lipid content [10,27,28]. In contrast to other fat-laden body organs, only a small lipid fraction is used as an energy source. The majority serve structural and signalling roles in the enormous expanse of membranes covering the organelles and surface of brain cells and their processes. Phospholipids account for approximately 50% of total lipids [29] and the other major contributors are cholesterol and its esters, sphingolipids, glycolipids, and fatty acids. Because of the blood–brain barrier (BBB), most of the cholesterol is synthesized de novo in the brain [215].

Due to liquid-liquid phase separation in cell membranes, lipids segregate into ordered (raft) and disordered (non-raft) domains [49,216,217,218,219,220,221]. Rafts are transient and dynamic, heterogeneous with an estimated diameter of 10–200 nm (average 50 nm), and enriched in sphingolipids, cholesterol, and lipids with saturated acyl chains. They harbour most of the proteins involved in synaptic transmission and the amyloidogenic secretases [34,222], and serve as a platform for cellular processes such as cell signalling, pathogen entry, cell adhesion, motility, protein sorting, and trafficking. Non-raft domains are enriched in unsaturated and polyunsaturated lipids and other subsets of membrane proteins [34,49,220,223,224]. Changes in the composition of membrane glycerophospholipids, cholesterol, and sphingolipid content impact the properties of lipid rafts, influencing signal transduction from membrane receptors and activity of membrane transporters [220,225].

### 5.1. Glycerophospholipids

#### 5.1.1. Synthesis

**Figure 6:**
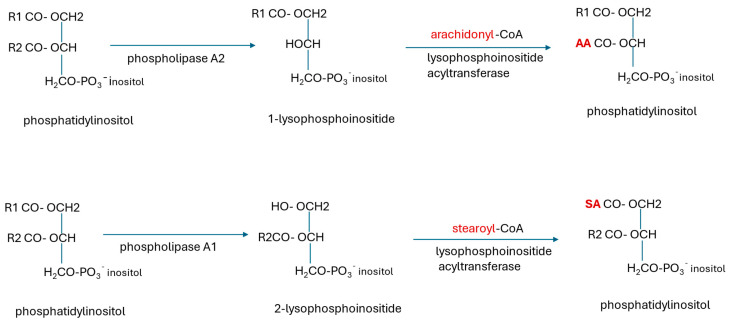
Remodelling of phosphatidylinositides by the Lands cycle. The phosphatidylinositides (PIs) synthesized de novo have a miscellany of fatty acyl groups. Phospholipase A1 or A2 removes the acyl chains at sn-1 or sn-2, respectively, leaving a lysophosphoinositide. The new, required acyl groups (shown in red) are transferred to this from stearoyl-CoA or arachidonyl-CoA. PIs incorporated into glycosylphosphatidylinositol (GPI)-anchored proteins are remodelled by the same process, but with replacement of flexible unsaturated fatty acyl chains at sn-2 with rigid stearate chains transferred from stearoyl-CoA [127,227]. Polyunsaturated acyl groups located at the sn-2 position have a rapid turnover. Synthesis, remodelling, and recycling of phospholipids are highly active processes with high ATP consumption [228,229]. AA arachidonic acid, SA stearic acid.

Glycerophospholipids are synthesized de novo mainly from glycerol-3-phosphate. In the initial step, GPAT1 (glycerol phosphate acyltransferase) acylates sn-1 with a preference for saturated stearoyl and palmitoyl fatty acids. Then AGPAT (acylglycerol phosphate acyltransferase) acylates sn-2 with a preference for oleoyl-CoA to form phosphatidic acid (1,2-diacylglycerol-3-phosphate) [226]. Further processing via the CDP-DAG (cytidine diphosphate-diacylglycerol) pathway yields glycosylphosphoinositols, phosphoinositides (PIs), phosphatidyl choline (PC), phosphatidyl ethanolamine (PE), phosphatidylserine (PS), and cardiolipin. CDP-DAG is a high-energy intermediate synthesized from cytidine triphosphate (CTP). The PI species and plasmalogens synthesized de novo have a miscellany of fatty acyl groups. They may be enriched with arachidonic acid (AA) or docosahexaenoic acid (DHA) by acyl chain remodelling via the Lands cycle (Figure 6) in the ER.

#### 5.1.2. Physiological Functions

Glycerophospholipids are essential to maintain membrane physical bilayer properties for the correct location (in rafts or disordered membrane) of integral proteins and their function. They supply arachidonic acid for eicosanoid production and phosphatidyl inositol PI 4,5 diphosphate, a key signalling molecule with rapid turnover. Approximately 50% of the total phospholipids of the inner mitochondrial membrane are composed of cardiolipin (CL) and phosphatidylethanolamine (PE) [230,231]. Their cone shape is essential for enabling curvature of the membranes and supporting the architecture of the mitochondrial cristae [231], which are the predominant site of OXPHOS assembly and operation [232]. CL also directly interacts with OXPHOS components and is required for the formation and stability of Complexes III and IV [233,234].

#### 5.1.3. Pathophysiology

##### Membrane Peroxidation of PUFAs

Lipids containing carbon-carbon double bonds, particularly polyunsaturated fatty acids (PUFAs), undergo free radical attack by oxygen radicals (ROS). The major cell sources are superoxides generated as byproducts of oxygen consumption at the mitochondrial respiratory chain [235]. Other sources include the activities of NADPH oxidase, cytochrome P450 enzymes, and 5-lipoxygenase [90]. ROS may be generated non-enzymatically, for example, by the Fenton reaction in which hydrogen peroxide (H_2_O_2_) reacts with Fe^2+^, or Cu^+^ [49,236,237,238], or the Haber–Weiss reaction in which superoxide interacts with H_2_O_2_ or another peroxide. Importantly, free radical attack on PUFAs in membranes initiates a self-perpetuating oxidative cascade which generates lipid hydroperoxides, thus propagating a rapidly spreading chain reaction [31,239]. The hydroperoxides disrupt membrane function by increasing membrane permeability and perturbing lipid packing, particularly in the disordered membrane regions which have a high PUFA content and hence alter protein distribution between these domains and rafts [49]. In addition, their degradation produces highly reactive aldehydes, 4-hydroxynonenal (4-HNE) from arachidonic acid, 4-hydroxyhexenal (4-HHE) from docosahexaenoic acid, acrolein, and malondialdehyde, which form adducts with lipids, proteins, DNA, and other biomolecules [10,49]. Damage to intracellular membranes, particularly of the ER, may activate the unfolded protein response [240]. Cells have a high capacity to mount rapid protective measures against free radical attack by recruitment of a host of enzymes including catalase, superoxide dismutase, glutathione peroxidase, and glutathione reductase, and non-enzymatic antioxidants such as glutathione, ubiquinone, uric acid, and thioredoxin [90,230]. Mitochondrial thioredoxin (TXN2) is expressed ubiquitously, with the highest expression in the brain [241] and operates in the mitochondrial thioredoxin system comprising nuclear-encoded peroxiredoxins 3 and 5 (PRDX3 and 5), thioredoxin 2 (TXN2), and thioredoxin reductase 2 (TXNRD2). TXN2 deficiency manifests as an infantile-onset neurodegenerative disorder [230]. Lipid peroxidation is counteracted by several repair systems, especially the system xc^−^/glutathione/glutathione peroxidase 4 (GPX4), ferroptosis suppressor protein 1 (FSP1)/CoQ10, and GCH1/BH4 pathways [242]. Ferroptosis is a form of non-apoptotic cell death that results from excessive iron-catalysed peroxidation of membrane phospholipids [243,244] and may contribute to cell death in degenerative diseases, including AD, and acute brain injury [90,242]. Susceptibility to ferroptosis is increased by the enrichment of phosphatidylinositol with arachidonate and eicosapentaenoate [242,245].

#### 5.1.4. Potential Role of Disordered Membrane Phospholipids in Promoting Aβ Production from Amyloid Precursor Protein (APP)

**Figure 7:**
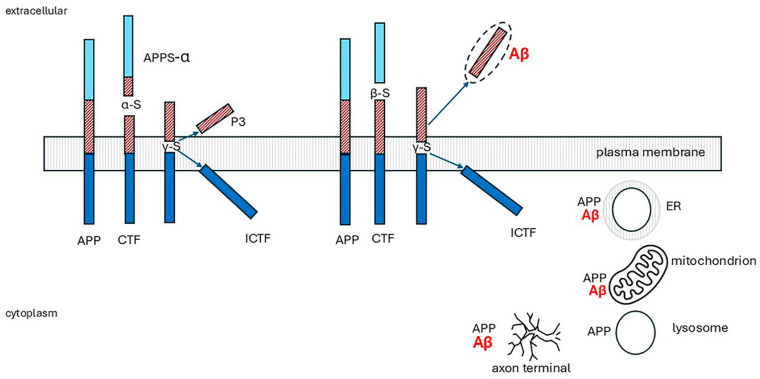
Processing of amyloid precursor protein (APP), not drawn to scale. Full length APP has three components: a large extracellular domain (light blue and brown) representing around 85% of protein mass, a short transmembrane sequence (brown and dark blue) and a small cytoplasmic domain (dark blue). APP is first cleaved extracellularly. In the non-amyloidogenic pathway (left), α-secretase (α-s) releases a soluble peptide, APPs-α. The 83-residue membrane-bound fragment is then cleaved by γ-secretase (γ-s) in its transmembrane domain releasing a soluble N-terminal fragment, P3 peptide and an intracellular C-terminal fragment (ICTF). In the amyloidogenic pathway (right), β-secretase (β-s, β-APP-cleaving enzyme 1, BACE) cleaves APP at a more proximal site, releasing an N-terminal peptide (light blue). The 99-residue membrane-bound fragment is then cleaved by γ-secretase, possibly at more than one site. ICTF is released into the cytoplasm; Aβ peptides with 39 to 42 residues are discharged extracellularly. They may aggregate, oligomerize, and form fibrils [34,49,249].

The physiological roles of APP are unknown, but proposed functions include regulation of neurite outgrowth, cell adhesion, synaptogenesis, and cell survival. APP knockout mice are viable but have impaired spatial learning and long-term potentiation [246]. There are three isoforms produced by alternative splicing: APP695, 751, and 770. APP695 is the major neuronal isoform. APP770 is expressed in most other cell types [34]. APP is synthesized in the endoplasmic reticulum (ER) and trafficked through the secretory pathway. Most is localized in the Golgi apparatus, trans-Golgi network (TGN), and post-TGN vesicles, and only around 10% reaches the plasma membrane. At the cell surface, APP is cleaved enzymatically or internalized into endosomes. APP is carried through neuronal axons via the anterograde transport machinery and may be the source of synaptically released Aβ [247,248]. APP is cleaved by two routes, a non-amyloidogenic pathway and the amyloidogenic Aβ peptide-producing pathway, shown in Figure 7 and explained in the legend. The non-amyloidogenic pathway predominates in non-neuronal cells.

The three secretases are transmembrane proteases. BACE1 is an aspartyl protease [250], α-secretase activity is associated with at least three members of the ADAM (a disintegrin and metalloprotease) family (ADAM9, ADAM10, and ADAM17) [251], and γ-secretase is a complex comprising four core subunits: presenilins (PS1 or PS2), nicastrin, PEN2 (Presenilin Enhancer 2), and APH1 (Anterior Pharynx defective 1) [252]. PS1 is the catalytic subunit. Intracellularly, BACE1 and γ-secretase are present in the TGN and endosomes. In cells transfected with APP, Aβ is mainly generated in these organelles. If this also occurs physiologically, it raises the question of the physiological role of APP at these sites [34]. There is good evidence that APP and C99 localize preferentially in the disordered region of cell membranes. Further, both were shown to remain exclusively associated with disordered regions of the membrane following lipid peroxidation [249,253]. The subcellular location of the enzymes is less certain. Variation in bilayer thickness, membrane ordering, and specific interactions with cholesterol affect the structure and orientation of the BACE1 and ADAM10 transmembrane domains. BACE1 can adapt more readily to the wider organized (raft) membranes than ADAM10, suggesting that ADAM10 may be less suited for localization in these domains than BACE1 [253]. γ-Secretase subunits were shown to reside in cholesterol- and sphingolipid-rich detergent-resistant lipid raft microdomains of post-Golgi, TGN, and endosome membranes. Both secreted and intracellular Aβ were significantly reduced in neuronal cells when cholesterol transport from late endocytic organelles to the ER was blocked by the cholesterol transport inhibiting drug, U18666A [254]. Similarly, increased cholesterol efflux mediated by ATP-binding cassette transporter A1 (ABCA1) decreased Aβ production by reducing BACE1 and γ-secretase cleavage of APP [255]. BACE1 has many other substrates [256], including low-density lipoprotein receptor-related protein (LRP), β subunits of voltage-gated sodium channels, interleukin-1 receptor II (IL-1R2), and neuregulin 1 and 3 [34]. If disordered membrane lipids decrease BACE1 activity, would this lead to the accumulation of products from its other substrates as well as Aβ which might be implicated in AD pathology?

#### 5.1.5. Disturbances of Membrane Lipids in AD

Studies of the brains of patients with AD have observed differences in the lipid content from unaffected controls [10]. Concentrations of PUFAs in membrane lipids have been lower in AD compared with controls, including the hippocampus and the entorhinal cortex. Low levels of glycerophospholipids, including phospholipid-bound arachidonate, sphingomyelin, and the myelin constituents galactosylceramide and sulfatides, were observed from the early stages of AD [10,257,258]. The low levels of PUFAs in membrane phospholipids in AD may be explained by decreased synthesis, increased release from sn-2-bound PUFAs, notably arachidonate, by phospholipase A2 which was reported to be increased in AD [259], inadequate replacement by the Lands cycle (Figure 6), or lipid degradation by ROS. In vitro, ATP depletion resulting from *slc25a25*-knockdown in renal cells had a dramatic effect on intracellular lipids, with increases in unbound PUFAs, lysoplasmalogens, and some phospholipids, and decreases in intermediates of phosphatide synthesis, and some lysophospholipids [85] (Section 3.2). These changes could have resulted from a combination of the factors enumerated above.

Single-nucleotide polymorphisms (SNPs) of genes involved in lipid turnover associate with AD. These include ATP-binding cassette subfamily A members 1 and 7 (*ABCA1* and *ABCA7*). ABCA1 initiates the efflux of lipids such as cholesterol and phospholipids by loading them onto lipid-free lipoproteins. A loss-of-function mutation in *ABCA1* is associated with an increased risk for AD [19]. ABCA7 is also involved in the transport of cholesterol and phospholipids. Multiple loss-of-function variants of *ABCA7* have been found to associate with altered lipid and Aβ metabolism and increased AD risks [20,21]. An SNP for the gene for sterol regulatory element binding protein-2, *SREBP-2*, which regulates cholesterol synthesis, is associated with biomarkers for AD [22].

## 6. Hypoperfusion of the Hippocampus

Many brain imaging studies have reported decreased cerebral blood flow (CBF) in patients with AD [260,261,262,263,264,265,266,267]. Reduced glucose uptake and perfusion in the hippocampus, parietotemporal cortex, and/or posterior cingulate cortex have been demonstrated by FDG-PET in AD in individuals with early AD, MCI, or no cognitive impairment prior to progression to AD [264,268,269], and in individuals at genetic risk for AD [270,271]. The primary problem is an inadequate blood supply and not reduced metabolic demand [262]. For decades, the blood supply to the hippocampus has been considered parlous, with limited capacity to meet increased demands [272,273,274]. When coupled with pathological dysfunction of arteries supplying blood to this region, for example, atheroma, hypertension, or vasospasm after subarachnoid haemorrhage [36,264,275,276] causing arterial constriction, there is a risk of local ischaemia during high neuronal activity. This could trigger a cascade of biochemical events contributing to AD, as described later in the Discussion (Section 8).

Comparison of the vasculature of the brain cortex and hippocampus in vivo using neuroimaging has explained the vulnerability.

### 6.1. Blood Supply to the Brain Cortex and Hippocampus

The brain’s blood supply is provided by three large arteries, the posterior, anterior, and middle cerebral arteries, that arise from the Circle of Willis, an arterial hub at the base fed by blood from the internal carotid and vertebral arteries. Those supplying the cortex branch into large pial arteries which run along the surface of the brain, become progressively smaller, and penetrate perpendicularly into the brain substance giving rise to arterioles and capillaries [36,264,272,277]. There are layers of contractile muscle cells in the walls of the arteries and arterioles. In the capillaries, these are replaced by pericytes, small smooth muscle cells which underlie the vascular endothelium and are enclosed within the basal lamina. Astrocytic end feet encase this basal lamina. The astrocytes, pericytes, endothelium, and adjacent neurons associate as a neurovascular unit. The pial arteries receive innervation from peripheral nerves, whereas arterioles and micro vessels are innervated intrinsically within the brain substance. Cerebral blood flow (CBF) varies by brain region and adapts constantly to ensure energy supply and waste removal to meet local needs. CBF increases during neuronal activity through the dilation of local arterioles in response to the concerted actions of a range of vasoactive agents produced by vascular cells, neurons, and astrocytes. Notable among these are nitric oxide, prostacyclin, adenosine, and K^+^ ions [36,264,278,279,280].

### 6.2. Features of the Hippocampal Vasculature Increase the Risk for Hypoperfusion

The source of blood for the hippocampus is variable. High-resolution 7 Tesla time-of-flight MR was used to visualize the brain vasculature of healthy young adults aged 19–34 years [272] The most common source (50% of hemispheres) was a combination from the posterior cerebral artery (PCA) and the anterior choroidal artery (AChA), in agreement with 57% found in an autopsy study [277]. The least common source (3–5%) was the AChA alone. Blood in the PCA was mostly from the vertebrobasilar artery and not the carotid arteries which are the source in small mammals. Different distribution patterns of the right and left hemispheres were observed [272]. Table 3 summarises studies to investigate CBF. Supplementary Table S1 includes experimental details.

Properties of the hippocampal blood vessels which would increase susceptibility to hypoxia and ischaemia are (i) the arteries and veins in the hippocampus have a long tangential course and few anastomoses [36,272], (ii) compared with the cortex, there are far fewer capillaries, and these are more widely spaced, and (iii) they are extremely narrow. The mean diameter of intrahippocampal arteries is 0.09 mm [272]. Delivery of oxygen and glucose may be compromised by low capillary density and red blood cell velocity [272,273,293].

These anatomical features are not confined to individuals with AD but apply to the whole population. Hippocampal perfusion is reduced in healthy older adults aged 60–77 years without dementia (Table 3 [283]). In individuals aged around 70 y, greater blood flow to the hippocampus was positively correlated with memory performance [36]. The vascular reserve of the hippocampus is now considered a primary contributing factor to cognitive performance [294]. Cardiovascular disorders [295,296], and conditions causing chronic hypoxia [297,298] are risk factors for sporadic AD. Vascular dysfunction is a prominent and early feature in prodromal AD [264]. Atherosclerosis of cerebral arteries or hypoxia (Table 3 [281,282]), or increased intra-arterial pressure in hypertension (Section 6.4), vasospasm following a subarachnoid haemorrhage [32,268,269] could all reduce the hippocampal blood flow to levels below the safety threshold. At autopsy, atherosclerotic stenosis was significantly greater in the circle of Willis arteries and large leptomeningeal arteries of individuals with AD than from non-demented controls [281,282]. The number of stenoses and the index of occlusion were positively correlated (R = 0.67; *p* < 0.00001), and the index of stenosis correlated with the scores for total amyloid plaque, neuritic plaque, neurofibrillary tangle, Braak stage, and white matter rarefaction. Severe stenotic lesions consisting of long and continuous stretches of atheroma plaque causing total arterial occlusion were observed in some arterial segments in the AD cases.

### 6.3. Neurovascular Coupling and the Effects of Hypoxia

The brain is protected from damage due to transient mild oxygen insufficiency during increased neuronal activity by a process termed neurovascular coupling. The neurons signal to local capillaries to dilate, thereby increasing local blood flow and oxygen and glucose delivery. The mechanism which mediates this response is unclear, but HIF may have a central role [36,273,299]. Severe episodic or sustained hypoxia initiates a cascade of pathological events that leads to neuronal degeneration [299,300,301,302].

Shaw, Bell, Boyd et al. [273] investigated neurovascular coupling in vivo in the mouse brain cortex through an implanted cranial window. Hippocampal (HC) blood vessels had a blunted response compared with those in the visual cortex (VI), with fewer, smaller dilations. Dilations of HC vessels larger than 7 µm were only half those in the V1. The calculated rate of O_2_ consumption (VO_2_) indicated that production of ATP through oxidative phosphorylation in the HC was restricted in the tissue furthest from a capillary. A low O_2_ concentration was estimated to decrease consumption by at least 10% in 30% of HC tissue, and by at least 20% in 10% of tissue. Shaw et al. surmised that, since O_2_ levels are limiting under physiological conditions, further decreases in O_2_ availability, as with decreased CBF or local brain ischaemia, would produce a greater reduction in ATP synthesis over a larger volume of tissue in the HC than in the neocortex.

**Figure 8:**
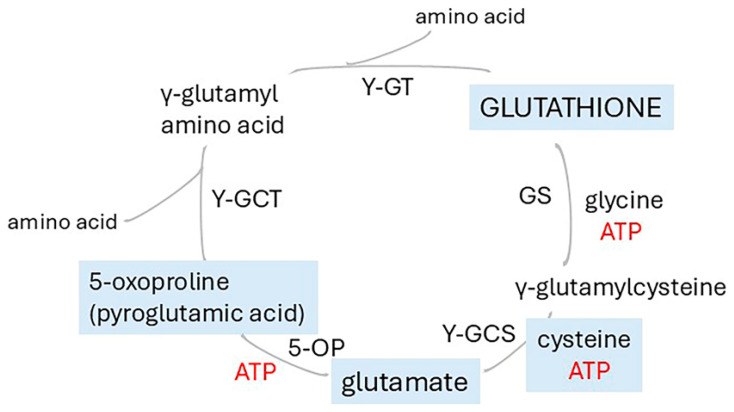
The γ-glutamyl cycle 5-oxoproline (pyroglutamic acid) is an intermediate in glutathione metabolism. The tripeptide glutathione is synthesized and degraded in the γ-glutamyl cycle. It is synthesized from glutamate by the sequential actions of γ-glutamylcysteine synthetase (γ-GCS) and glutathione synthetase (GS). γ-glutamine transpeptidase (γ-GT) initiates breakdown by transferring the γ-glutamyl group to amino acid acceptors. γ-glutamylcyclotransferase (γ-GCT) forms 5-oxoproline and releases the amino acids. Notably, 5-oxoprolinase (5-OP) converts 5-oxoproline to glutamate. A dipeptidase (not shown) splits the cysteinylglycine moiety of glutathione into cysteine and glycine. The cycle has heavy ATP consumption.

In healthy young men, hypobaric hypoxia (12% O_2_) reduced blood flow to brain regions with roles in memory functions (Table 2). Hypoxia for approximately 7 h significantly reduced O_2_ delivery of the middle cerebral artery during an executive task and of the posterior cerebral artery during memory tasks but had no effect on cognition [274]. Hypoxia for 2 h decreased perfusion in regions of the brain normally associated with the ‘default mode’ or ‘task negative’ network. The decrease resulted from vasoconstriction. After 10 h of hypoxia, decreased flow to the default mode network was more pronounced and widespread, involving the posterior cingulate and cuneal cortex, which have roles in declarative and procedural memory [285]. Significantly, decreased flow to the cingulate gyrus was reported in hypertensive individuals (Table 2 [287]). Ischaemia led to widespread biochemical disturbances in the human brain ex vivo and the brain from a mouse stroke model (Table 2 [286]). Notably, there was a large increase in succinate, but the other TCA intermediates decreased. ATP levels fell dramatically, which probably explains the observed large increase in 5-oxoproline, an intermediate in the biosynthesis of glutathione (Figure 8). A fall in glutathione production would dramatically reduce protection from ROS.

Exposure of rodents to hypobaric oxygen also had a significant metabolic impact on the brain (Table 2 [289,291]). The hippocampus was most susceptible [291]. Energy production was disturbed (increased lactate dehydrogenase and glutamate dehydrogenase), and ROS generation increased significantly and progressed with time. Protection from ROS decreased strikingly (Table 2 [289,291,292]). The disturbances increased with the duration of hypoxia and were more evident in the hippocampus than in the brain cortex. Exposure of rats to hypoxia for 4 days led to significant morphological changes in the hippocampus. The damage increased between 72 h and 144 h after hypoxia, suggesting delayed neurotoxicity (Table 2 [290]).

Collectively, the studies demonstrate that hypoxia causes a significant decrease in hippocampal blood flow. This causes widespread biochemical disturbances with free radical generation which increases with the duration of hypoxia and may cause delayed neurotoxicity.

### 6.4. Effects of Hypertension on Cerebral Blood Flow

Systemic arterial pressure varies during normal activities. The brain self-protects against these oscillations by a process of autoregulation in which the resistance of the cerebral arterioles is adjusted according to intravascular pressure [36,264]. By constricting as pressure increases and relaxing as it decreases, the arterioles normally maintain a constant cerebral blood flow over the range 60 to 150 mm Hg. This is mediated by depolarization of arterial muscle cells by rising intraluminal pressure, resulting in an influx of Ca^2+^ and vasoconstriction, together with actions of protein kinase C and Rho kinase which increase the muscle contractile response. Endothelium-derived constrictor factors participate in the maintenance of basal arterial tone. Vasodilation is regulated by the release of NO, prostacyclin, bradykinin, and other agents by endothelial cells [32,36,280].

Hypertension causes changes in arterial structure which can impair blood flow, especially during ischaemic insults or hypotensive episodes. Sustained increases in intraluminal pressure stimulate remodelling of the arterial muscle cells which may undergo hypertrophy and hyperplasia causing thickening of the arterial wall, or rearrangement. Both mechanisms lead to narrowing of the arterial lumen. Arterial resistance is inversely proportional to the luminal diameter, which therefore determines blood flow [36]. In hypertension, myogenic activity (autoregulation) operates at a higher range of pressure (approximately 65 to 190 mm Hg) in cerebral arteries [279]. In addition, hypertension may cause arterial stiffening, promote atherosclerotic plaque formation, or cause fibrinoid necrosis of the penetrating arteries which can lead to white matter infarcts or small haemorrhages [303]. The BBB is impaired, with loss of brain protection [36,264]. In the stroke-prone, spontaneously hypertensive rat (SHRSP) model, remodelling increases with age, and the lumen diameter decreases progressively [279]. A variety of factors contribute to muscle hypertrophy, including trophic effects of sympathetic nerves, mechanical effects of increased pressure, increased growth factors, AII (angiotensin II), and oxidative stress [36,264]. Impaired endothelial-mediated vasodilation and regulation of myogenic activity compound the arterial dysfunction. Contributory factors include increased ROS generation via NADPH oxidation, decreases in superoxide dismutase (SOD) and cystathionine b-synthase, deficiency of NO (nitric oxide) due to inactivation by ROS and to decreased activity of eNOS (endothelial nitric oxide synthase), decreased endothelium-derived hyperpolarising factor (EDHF), decreased vasodilatory eicosanoids, prostacyclin, and epoxyeicosatrienoic acids, and impaired function of ion channels including store-operated calcium channels, calcium-activated K^+^ channels, and transient receptor potential vanilloid channel 4 (TRPV4) [32,36,280]. Collectively, they decrease basal CBF and functional hyperaemia to support increased brain activity.

In a longitudinal study, regional CBF in prefrontal, anterior cingulate, and occipital areas decreased over 6 years in older individuals with hypertension compared with controls (Table 2 [287]). Hypertension in midlife increases the risk of AD in later life. It accelerates the progression of AD and is associated with an increase in amyloid deposition [260]. It probably precedes the development of plaques. From observations in SHRSP rats, arterial narrowing may progress with age without effective blood pressure control [304]. Early diagnosis of hypertension and good control are now regarded as priorities for reducing the risk of AD.

## 7. Genomic, Proteomic, Metabolomic, and Imaging Investigations to Identify Causative Genes and Pathways in AD

### 7.1. Human Studies

The number of studies to explore the genomics of AD using the advanced analytical and information technology now available has escalated. Recent focus has been on identifying disturbances in molecular pathways, rather than associations with individual compounds, in order to obtain a broader view to aid reconstruction of events. Many have analysed brains or data from carefully collected samples from cohorts of individuals with AD and controls with normal cognition. The analytical results must be interpreted with caution because of well-recognized problems with the samples analysed. Brain chemistry changes very rapidly ex vivo, and this must also be true of the transcriptome which may not give a true picture of the in vivo situation, despite comparison with controls. Metabolites in CSF reflect production across the whole brain surface and small, deeply seated regions are poorly represented. Further, normally about 80% of the total protein amount in CSF derives from size-dependent filtration of blood across the blood–brain barrier (BBB) [305]. The human studies documented in Table 4 and presented with more detail in Supplementary Table S2 were selected because they included data for the hippocampus or entorhinal cortex, and/or for early stages of AD, and/or investigated metabolic pathways relevant to the proposal under investigation. Studies of the brain rather than CSF and blood were preferred, first because the search was for localized early changes in the hippocampus, and second because studies using CSF Tau and Aβ amyloid as inclusion criteria for AD were probably investigating a relatively advanced stage of the AD progression. Readers should refer to the papers reporting the genomic studies. They present a wealth of data which the tables here cannot convey.

Because the objectives and the research procedures differed, it is impossible to group the data. Of the 13 studies tabulated which analysed brain metabolites, six identified significant differences in OXPHOS, mitochondrial function, or energy pathways compared to controls (study number [Ref]: 3 [16], 6 [47], 7 [308], 11 [311], 13 [313], 15 [2]). This is consistent with the growing view that mitochondrial dysfunction is a major pathogenic factor in AD. Of interest, Study 3 [16] demonstrated increased expression of OXPHOS subunits in young asymptomatic *APOE4* carriers, and Study 15 [2] observed increased expression in individuals with AD pathology but normal cognition (AD-resilient) compared to elderly non-demented individuals. This might reflect responses to ATP depletion (refer to discussion in Section 8). Other observations from the brain studies to highlight are Studies 1 [1] and 4 [38], which demonstrated that neuronal cell numbers and hippocampal volume are maintained in older individuals with normal cognition in contrast to AD and Study 2 [306], which observed marked disturbances of the malate-aspartate shuttle, glycerophospholipids, and pyrimidines in the brains of individuals with AD within 4 h of death. These are similar to findings for ATP-depleted renal cells with SLC25A25 deficiency [84,85] (Section 3.5 and Section 5.1.5). Study 10 [310] observed decreases in branched-chain amino acids with AD progression, probably explained by increased catabolism for energy. Medium- and long-chain acylcarnitines were increased, which was a surprising finding. These are diagnostic markers for deficiency of very long-chain fatty acid dehydrogenase (VLCAD), which is located at the inner mitochondrial membrane (IMM). Reduced enzyme activity might reflect IMM damage. In contrast, the expression of the gene for carnitine palmitoyltransferase A (CPTA1) was increased. This enzyme, on the outer mitochondrial membrane, converts acyl-CoA (long-chain fatty acids) into acylcarnitines for transfer into mitochondria. Study 13 [313] found that the expression of 88 proteins was needed to predict the progression of asymptomatic AD to AD. Study 14 [18] identified associations of three mitochondrial solute-linked carrier 25 (SLC25) carriers with AD. One, SLC25A22, which codes for glutamate carrier 1, was proposed to participate in the glutamate/GABA/glutamine cycle.

Three of the four CSF studies, 18 [315], 19 [316], and 20 [317], reported the association of enzymes involved in glucose metabolism with AD. Preliminary observations suggested that dysregulation of glucose metabolism may precede impairment of cognition [317].

### 7.2. Animal Studies

Because of the problems of investigating AD in humans, animal models have a central role in AD research. Table 5 summarises studies of brain or CSF which have measured metabolites.

A problem highlighted with transgenic AD models is that they resemble early-onset genetic AD rather than the common late-onset disorder (LOAD) [26]. Non-transgenic rabbits fed a high cholesterol diet have amyloid deposits and other pathological markers and resemble LOAD more closely. Differences in CSF metabolites from controls were observed in rabbits on a high cholesterol intake for 3 to 12 weeks, with most of the abnormalities at 12 weeks. The majority were glycerophosphates and phosphorylated fatty alcohols. Aβ-like plaques were only observed at 12 weeks (Study 21 [26]). Decreases in TCA intermediates and oxidative glutamine were recorded in the cortex and hippocampus from APP_swe_/PSEN1dE9 AD mice aged 3m, before amyloid plaque development 22 [318]. Marked mitochondrial dysfunction was evident in the hippocampus and frontal cortex from transgenic 25476 AD mice, a commonly used AD model (Study 27 [323], affecting energy-generating mitochondrial pathways including OXPHOS pathways and fatty acid oxidation. From gene set enrichment analysis (GSEA), oxidative phosphorylation was the most down-regulated gene set in the hippocampus of early symptomatic Tg2576. An NH2htau antibody produced significant resolution of the abnormalities. In a third mouse model (Study 25 [321]) expressing 4-42 Tau which is truncated at the N-terminal, significant metabolic differences from controls were observed in the caudate and putamen regions at 9 m of age. Aβ accumulated intracellularly, but there were no plaques. These mice develop cognitive deficits. Notably, 4-42 Tau is not produced physiologically and is more toxic than Aβ1-40 and Aβ1-42. Study 24 [320] demonstrated that the proteomes of repetitive mild traumatic injury to the brain and of an AD mouse model showed little overlap, indicating different mechanisms for brain damage.

## 8. Discussion

**Figure 9:**
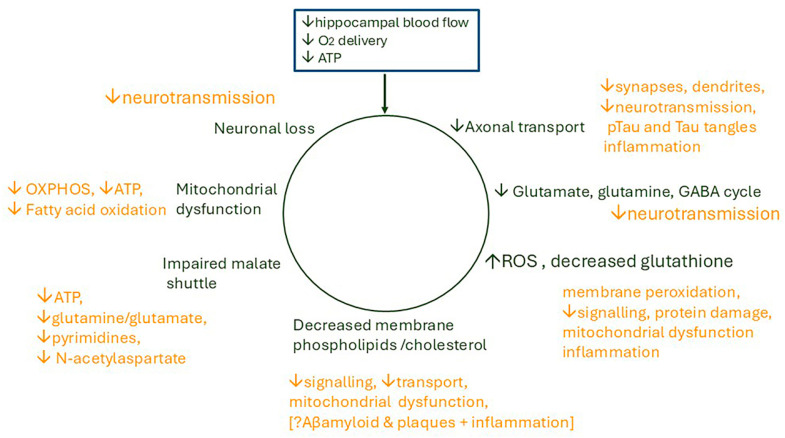
The impact of low hippocampal blood flow on neuronal activities. It is proposed that reduction in blood flow, causing poor perfusion of the hippocampus, reduces oxygen delivery to the nerve cells, and ATP synthesis falls. This disrupts essential neuronal activities (shown in dark green) precipitating a cascade of biochemical disturbances (shown in orange). Repeated episodes cause cumulative damage and inflammation which slowly spreads further into the brain and impacts memory and learning. ↓ decreased; ↑ increased; ? speculated may be implicated in Aβ and plaque formation.

The proposal under consideration is that inadequacies of the blood supply to the hippocampus lead to clinically silent focal episodes of hypoperfusion. The resulting ATP depletion disrupts essential neuronal activities, causing a cascade of cumulative damage and inflammation which slowly spreads further into the brain and impacts memory and learning (Figure 9).

This seems a feasible, neat, and logical explanation to explain the onset of Alzheimer’s disease. The brain is wholly reliant on a continuous supply of ATP to function, and loss of supply can be predicted to have widespread consequences, particularly on processes with the heaviest ATP consumption. The high content of PUFAs in membrane phospholipids provides an abundant substrate for cascading free radical attack causing peroxidative damage, which is a common feature of brain ischaemia. This would affect signalling and transport functions throughout the cell and provoke an inflammatory response at the cell surface. An individual with an unfavourable hippocampal vascular supply would be predicted to have numerous minor ischaemic events over the years whenever the ‘head pressure’ of the main arterial supply falls. These would become more frequent after middle age with cerebral arterial narrowing due to atheroma and/or hypertension, and result in cumulative spreading damage. It is an attractive proposition, but is it tenable? Examination of the scientific basis for ATP depletion and its likely consequences demonstrates that it could indeed have major consequences on mitochondrial and membrane functions, biosynthesis and recycling of glutamate neurotransmitters, and axonal transport of organelles. It might possibly explain the accumulation of Aβ amyloid and plaque development, and Tau tangles. Excessive ROS activity would make a significant contribution to the cascading cell damage. The case for hypoperfusion rests on the parlous blood supply to the hippocampus resulting from variations in its vascular supply, and/or restricted blood flow through narrowed blood vessels. Findings from recent genomic studies are disparate, reflecting differences in the study objectives. None of those considered here presented evidence which conflicts with the proposal (Table 4), however, some reported abnormalities which would favour alternative mechanisms as driving forces, notably in inflammatory pathways. Collectively there is support for the proposal (Section 7.1) which may open new avenues for prevention/intervention. It is interesting that a study of the brains of individuals with Parkinson’s disease found that critical processes affected in early disease, before synucleinopathy and neuronal loss, were oxidative phosphorylation and ATP synthesis and glutathione, and redox regulation [324].

This is an extensive subject, and only the most significant points can be addressed here.

Provision of a ready supply of ATP is paramount for normal brain function. The requirement for ATP is monitored continually by sensors (S2.2) which react immediately to a range of molecular changes, notably a decreased AMP/ATP ratio (AMPK), increased NAD^+^/NADH ratio (sirtuins), decreased phosphofructokinase activity (fructose bisphosphate), and hypoxia (HFI1 and HUMMR), which indicate a threat to supply. They take corrective action, increasing transcription of numerous genes to activate pathways to increase ATP synthesis, reduce energy expenditure, and increase the supply of oxygen and/or metabolic substrates. The generation of appropriate amounts of ATP must be controlled by the suppliers—namely the mitochondria. This necessitates very close co-ordination of mitochondrial and extramitochondrial activities, particularly to govern the profile of transcribed genes and to continually adjust the production of proteins to metabolic responses. Discovery of the mitochondrial-derived peptides (MDPs) over the last 15 years has been an exciting development which provides some insight into how this is achieved (Section 3.4). Although their function is still not well understood, it is of great interest that four have been shown to impact mitochondrial respiration. Humanin and SHLP2 increased respiration, and SHLP2 had a protective interaction with mitochondrial complex 1 [17]. MOTSc, however, decreased mitochondrial respiration and increased glycolysis in vitro and MtALTND4, which decreases complex 1 activity, reduced the O_2_ consumption rate and maximum coupled and uncoupled respiration [103]. There are probably other MDPs to be discovered. They are likely to be key regulators of mitochondrial function. Microproteins encoded within nuclear genes which modify mitochondrial function have also been identified. MIEF1-microprotein was reported to be involved in mitochondrial fission [105] and mitoregulin to support super complexes and to modify mitochondrial respiratory efficiency [99]. These findings indicate intense cross-communication between the mitochondria and nucleus which likely shapes the responses to significant hypoxic disturbances.

The malate-aspartate shuttle (MAS), glutamate/GABA/glutamine cycle, and axonal transport of organelles and proteins, which have a very high ATP turnover, are activities at high risk of disturbance with reduced energy supply (Section 4). The striking feature is the symbiosis of neurons and astrocytes in these processes. Future studies should perhaps investigate them as a single unit, possibly in organ cultures. In addition to essential roles in energy transfer and maintenance of redox balance in neurons, the MAS provides aspartate for export from neurons to adjacent astrocytes for glutamine synthesis and anaplerosis. In turn, astrocytes release glutamine to neurons for glutamate and GABA synthesis and as an energy source. Depletion of ATP, resulting in failure of these three processes, could have a major role in the loss of neurons, dendrites, and synapses; dystrophic axons and axonal swellings; as well as the reduced and disordered neurotransmission observed in AD (Section 1). Tau accumulation appears to be a consequence of reduced axonal trafficking along microtubules. This may be in response to signalling and kinase activation initiated by the neuronal nucleus to reduce energy consumption when ATP is low (Section 4.3). Phosphorylation of epitopes in the proline-rich domain and C-terminus of Tau leads to detachment of Tau from the microtubules, which are thereby destabilized and disaggregated. Released phosphorylated Tau may form tangles (NFTs) in the cytosol. Tau tangles induce an inflammatory response in nearby microglia which adds to AD damage. However, Tau has additional functions in axonal transport. It binds to histones in nuclear DNA and may be involved in chromatin remodelling and possibly protection of DNA from damage. This may be relevant to individuals with normal cognition who express Tau.

ATP depletion would be predicted to cause disturbances in brain lipids. Synthesis and remodelling of lipids have high energy dependency (Section 5). Deficiencies of phospholipids result in disordered lipid distribution in cell membranes. Increases or decreases in membrane cholesterol affect the compaction of lipid rafts and the location and activities of membrane-bound proteins and other molecules. These disturbances impact signalling. Phospholipids (cardiolipin and phosphatidylethanolamine) have an essential structural function in inner mitochondrial membranes by enabling membrane curvature and providing support for mitochondrial cristae which are the predominant sites of OXPHOS assembly and operation, and other super complexes. However, on the downside, the high content of PUFAs in brain membranes is a rich substrate for cascading free radical attack, causing unregulated propagation of damaging lipid peroxidation. This is a common feature of brain damage, particularly with ischaemia. Normal brain cells are well equipped to counteract this with protective antioxidant proteins and enzymes. Studies of the brains of patients with AD have reported differences in the brain lipid content from unaffected controls, evidence of increased ROS activity, and decreased protective agents (Section 5.1 and Section 7). Changes in membrane glycerophospholipids were observed in early AD. Loss-of-function polymorphisms in two genes involved in cholesterol and phospholipid turnover, ATP-binding cassette subfamily A members 1 (*ABCA1* and *ABCA7*), are associated with AD (Table 1). ABCA1 initiates cellular efflux of lipids by loading them into lipid-free lipoproteins. Is it possible that ApoE4 is among these, and, if so, could this explain its association with AD?

Amyloid Precursor protein (APP) (Section 5.1.4.) is a transmembrane protein which is mainly located in intracellular organelles, the Golgi apparatus, trans-Golgi network (TGN), and post-TGN vesicles, with only around 10% at the cell surface. Processing by transmembrane enzymes β-secretase (BACE) and γ-secretase produces Aβ1-40 and Aβ1-42 amyloid which form amyloid plaques. It is widely held that the accumulation of soluble Aβ peptides is the main causative event triggering the pathogenesis of Alzheimer’s disease. This is in apparent conflict with the proposal here, although the increased generation of Aβ peptides requires explanation [27,28] and the hypoxia proposal is one possibility. Queries to raise are, first, could misalignment of APP and the cleavage enzymes in ROS-damaged membranes divert APP to the amyloidogenic β-secretase pathway and away from the non-amyloidogenic α-secretase pathway (Figure 7)? Second, since BACE1 and γ-secretase are present in the TGN and endosomes, do APP and the Aβ peptides have roles in these organelles? Third, BACE1 has many other substrates. Is it possible that abnormal processing of one or more of these proteins could yield damaging peptides?

The number of studies exploring the genomics of AD using the advanced analytical and information technology now available has escalated. It was necessary to restrict those presented to studies providing data closely relevant to the proposal under review. The human studies enumerated in Table 4 (Section 7) had varying objectives and used different analytical approaches. Five of twelve brain studies which undertook genomic, proteomic, or metabolomic studies on individuals with AD identified significant differences in OXPHOS, mitochondrial function, or energy pathways compared to controls. This is consistent with the growing view that mitochondrial dysfunction is a major pathogenic factor in AD. These changes were not observed in all studies, however, and only three of the twelve studies found lipid disturbances, and one identified a marked disturbance in the malate-aspartate shuttle, while Studies 3 and 15 in Table 4 unexpectedly observed increased expression of respiratory chain components. This could indicate a response to a low energy status. If this is the case, there might not be enough reserve capacity to restore ATP levels should the blood flow to the hippocampus fall, or neuronal hyperactivity increase ATP consumption. Study 3 investigated young asymptomatic individuals heterozygous for *APOE4,* and Study 15, individuals with AD markers but normal cognition (AD resilience). It is clearly important to protect the brains of healthy elderly at times of risk, for example, following a cardiac or neurovascular event, surgery, or acute urinary infection, by careful control of oxygen intake, blood pressure, and prompt antibiotic and antioxidant administration. Short-term administration of protective drugs may be a future option. Cardiologists are exploring ways to prevent memory and cognitive loss after cardiac surgery [273]. Boosting the anaplerotic capacity of astrocytes would give longer protection. Triheptanoin has been used in clinical trials for neurological disorders (Section 4.2.4). Creatine phosphate to increase the energy reserve might be another possibility (Section 8.1.1 [325]). Young carriers of the *APOE4* allele have increased medial temporal lobe activity during active encoding tasks compared to non-carriers [326,327,328], which would increase ATP consumption. In addition, there is evidence that *APOE4* carriers are less able to regulate cerebral metabolism than *APOE4*-negative individuals [329]. Adding ApoE4 to well-being checks at middle age, perhaps with follow-up brain imaging to check for cerebrovascular abnormalities, would identify carriers for protective measures as outlined. Administration of an ApoE-mimetic peptide at times of increased risk (Section 8.1.1) may have therapeutic potential, as may creatine phosphate [325].

Reduced glucose uptake and perfusion in the hippocampus, parietotemporal cortex, and/or posterior cingulate cortex demonstrated by FDG-PET are early features in AD which may precede cognitive impairment [Section 6]. Largely because of recent advances in neuroimaging, this can now be explained by a variable combination of anatomical characteristics of the hippocampal vasculature and reduced blood flow to the hippocampus, most often because of narrowing of the supplying arteries (Section 6). Atheroma and hypertensive vasoconstriction are the commonest causes. The main source of blood in humans is the vertebrobasilar artery, but the arterial branches from this, which supply the hippocampus, vary and may differ between hemispheres. Recent in vivo studies of healthy young adults using time-of-flight angiography with very high resolution demonstrated that hippocampal arteries have few anastomoses and that, compared to the brain cortex, the hippocampus has relatively few capillaries and these are widely spaced. In addition, the protective vasodilatory response of hippocampal blood vessels to low blood O_2_ concentrations is blunted compared with cortical vessels [273]. O_2_ delivery to tissues furthest away from the capillaries will be most affected when delivery falls. Collectively, these factors explain why the hippocampus is vulnerable to hypoperfusion and hypoxia and why the vulnerability differs between individuals.

Although low O_2_ concentrations in the blood due to lung disorders and other hypoxic conditions reduce O_2_ delivery, by far the commonest cause is reduced blood flow through the narrowing of the lumen of hippocampal arteries. The association of AD with cardiovascular diseases has been clearly demonstrated. Arterial narrowing may be caused by atherosclerosis of the cerebral arteries. Although common in the population, in some individuals with AD, it may be more extensive than in non-affected subjects. The problem in hypertension is hypertrophy and hyperplasia of arterial muscle cells causing thickening of the arterial wall, sometimes with arterial stiffening, which increases the resistance to flow. Hypertension may also promote arterial atheromatous plaque formation, damage arteries perforating the brain substance, and decrease the blood–brain barrier. The arterial resistance increases with time, with a progressive decrease in the arterial lumen. Section 6.4 enumerates some of the many factors that contribute to muscle hypertrophy. Clearly to increase protection against the development of AD, treatments to prevent the progression of atheroma and to control blood pressure must be initiated early and monitored.

Subarachnoid haemorrhage (SAH, a bleed on the surface of the brain due to a ruptured brain aneurysm) leads to cognitive impairment in approximately 50% of patients [268,330]. In around 70% of patients, vasospasm of the cerebral arteries develops from the third day post-bleed, peaks after a week, and then subsides [32]. Of interest, expression of *APOE4* is associated with a higher risk of cognitive morbidity and delayed ischaemia following SAH [331,332]. In a rat model of SAH, administration of an ApoE-mimetic peptide improved functional outcomes and reduced evidence of vasospasm following SAH [333]. Vasospasm may be one of several factors that contribute to cognitive loss but it is thought not to be the sole or primary cause [268]. There is no reported association of SAH with AD. The association of vasospasm with ApoE is interesting and perhaps merits further exploration.

### 8.1. How Could the Hypoperfusion/ATP Depletion Model Influence Clinical Practice?

#### 8.1.1. Therapy

Investigations to find new therapeutic approaches for AD have escalated rapidly and become a field of intense activity. Two disease-modifying treatments with anti-amyloid monoclonal antibodies (mAbs) have been approved for use in the UK and by the FDA (U.S. Food and Drug Administration) for the treatment of early-stage AD: lecanemab and donanemab, although cost restricts their availability [334]. A third FDA-approved antibody, aducanumab, is no longer manufactured. Anti-amyloid mAbs reduce plaque burden, slow disease progression, and cognitive decline in patients with early symptomatic AD [335,336,337]. However, their overall effects on cognition are modest, and they have serious side effects (ARIA, amyloid-related imaging abnormalities consisting of vasogenic oedema or microhaemorrhages and haemosiderosis [336,337,338]). Remternetug (Eli Lilly, Indianapolis, USA), a second-generation immunotherapy drug that targets amyloid, is being investigated in trials in individuals with early-stage AD, and with mutations causing AD [334]. However, many new potential therapies adopting different approaches to tackle the disease are being explored, and some are already being evaluated in clinical trials [10,334,335,339,340]. In this context, only those interventions relevant to this review and currently under investigation or previously evaluated in individuals with AD are highlighted.

Benfotiamine is a synthetic vitamin B1 precursor shown to have many beneficial actions on AD pathologies in animal models [335]. Thiamine is an important coenzyme for transketolase, pyruvate dehydrogenase, and 2-oxoglutarate dehydrogenase, and hence essential for brain energy metabolism. In a phase 2a clinical trial, individuals with MCI or AD took benfotiamine or a placebo orally twice daily for 12 months. There was significantly less cognitive decline in the treated group than in the placebo group, and this effect was more potent in *APOE4* non-carriers. The effects were still present after one year of treatment, *p*-value: 0.002 [341]. A new 18-month trial is underway.

Creatine monophosphate. In AD mouse models, creatine monophosphate (CrM) supplementation improved cognitive function and brain energy metabolism and reduced amyloid beta (Aβ) and phosphorylated Tau [342,343]. In a recently reported single-arm pilot trial, 20 patients with AD received 20g/day of CrM for 8 weeks [325]. Serum creatine was elevated at 4 and 8 weeks (*p* < 0.001), and brain total creatine increased by 11% (*p* < 0.001). Cognition improved significantly in five tests, *p* values 0.02, 0.004, 0.001, <0.001 and *p* = 0.05. This could be a very useful observation. Further studies are indicated. Creatine phosphate is the ‘fall-back’ energy supply for depleted ATP, and the findings are consistent with the proposed role of ATP depletion in AD. Of interest, single synapse analyses of the hippocampus of individuals with AD and high Tau had significantly increased expression of the two enzymes that catalye creatine synthesis: glycine amidinotransferase (GATM, alias AGAT) and guanidinoacetate N-methyltransferase (GAMT) [344], probably indicating a drive for creatine production.

CMS121 is a small fatty acid synthase inhibitor derived from fisetin, a dietary antioxidant. It protects cells from cell death caused by glutathione depletion and inhibits lipid peroxidation and production of 4-hydroxynonenol in the hippocampi of AD mice. It has been evaluated in a phase 1 safety study and has Investigational New Drug (IND) approval [345].

CN105 is a novel ApoE-mimetic. Activation of microglial cells by brain injury initiates an inflammatory cascade which contributes to oxidative stress, secondary neuronal injury, blood–brain barrier breakdown, resulting in cerebral oedema, and tissue and cellular disruption. Glial secretion of ApoE increases after acute CNS injury and has neuroprotective actions [346]. CN105 is a pentapeptide which crosses the blood–brain barrier. Intravenous administration resulted in histological and functional improvement in various mouse models with acute and chronic brain injury [333,346,347,348]. CN105 is being tested in several phase 2 studies to evaluate efficacy after acute intracerebral haemorrhage and postoperative neuroprotection [346,349]. CN105 or other ApoE-mimetics might have particular value in the management of ApoE4 carriers.

ω-3 PUFA supplements: Small studies suggest potential beneficial effects of ω-3 PUFAs on pathological outcomes in MCI or AD patients [10]. Although two larger trials found that DHA had no effect on cognitive decline in subjects with mild-to-moderate AD, there was a positive effect in very mild AD [350] and in ApoE4 non-carriers [350,351].

Ketogenic diets (high saturated fats, low carbohydrates): Ketogenic diets provide an alternative energy source to glucose. Randomized controlled trials in patients with AD/mild cognitive impairment found that short-term therapy improved general cognitive function, daily function, and quality of life [352,353,354]. Long-term ketogenic therapy improved cerebral ketone uptake and utilization and episodic and secondary memory, but not psychological health, executive ability, or attention. The response in *APOE4* carriers was delayed [354].

Statins: Randomized clinical trials found no benefit of statin treatment in AD patients [10,355,356,357,358]. The reduction in AD risk, which was observed in observational studies, is proposed to result from the beneficial effects of lowering circulating cholesterol on the cardiovascular and cerebrovascular systems [359].

PPAR (Peroxisome Proliferator-Activated Receptor gamma) agonists: Although early trials with the PPARγ agonists pioglitazone and rosiglitazone looked promising, larger trials found that neither drug delayed the onset of MCI [10,360,361,362].

Acetyl-L-carnitine (ALCAR): improves age-associated mitochondrial dysfunction and cognitive deficit in animals. The effects on cognition in AD clinical trials were inconclusive [363].

Deferiprone. Several of the biological abnormalities seen in AD are consistent with free radical damage from impaired iron homeostasis. A placebo-controlled phase II trial reported that intramuscular injections of the iron chelator desferrioxamine slowed cognitive decline in subjects with AD by 50% over a 24-month period [364]. A recent phase 2 placebo-controlled trial found that oral administration of a less aggressive chelator, deferipone, for 12 months to patients with amyloid-confirmed MCI or early AD decreased hippocampal iron but accelerated cognitive decline. This suggested that lowering iron with deferiprone is detrimental to patients with AD [365,366]

Creatine, benfotiamine, and CMS121 all protect against the consequences of ATP depletion and appear to be potentially useful therapeutic agents. Ketogenic diets may provide some ancillary support. CN105 may help to reduce the inflammatory response to ischaemic brain injury and could have a particular protective role in *APOA4* carriers.

#### 8.1.2. Potential Markers

The search for markers to detect the very earliest evidence of AD in peripheral blood is being pursued intensively worldwide. Two new studies commenced in the UK this year in a joint venture. The READ-OUT (REAl world Dementia OUTcomes) study, based at the Universities of Oxford and Cambridge, will test multiple existing and novel blood tests for all stages of AD. At University College London, the ADAPT study (Alzheimer’s disease Diagnosis and Plasma pTau217) will focus on p-tau217 [367]. Data collected from studies of the molecular profiles in the brain and CSF from patients with AD are the starting point for the quests. Some of these studies are included in Table 4. It is evident that a single marker will not suffice (study 13 in Table 4 [313]). A strong marker in blood would be a compound which is not a normal blood constituent, or is a normal blood constituent but present at a clearly abnormal concentration (preferably raised), or present in an abnormal ratio with a related compound. Association with abnormal concentrations of other relevant metabolites would enhance its value. One unusual finding from the reviewed studies was the presence of succinylcarnitine in association with phosphorylated Tau in the CSF of AD subjects. This was not associated with amyloid in the absence of Tau and was replicated in an independent cohort [317]. Its source is unknown. Formation from succinyl-CoA in peroxisomes is one possibility [368]. It could be a potential marker and merits further investigation. The metabolite changes observed in ATP-depleted renal cells [84,85] highlight pathways likely to be disturbed if ATP depletion is a major factor in early AD, as proposed here, and if similar disturbances occur in brain cells. There was clear evidence of a significant disturbance of glutathione synthesis by the γ-glutamyl cycle (Figure 8), with increases in oxidized glutathione, increased γ-glutamyl branched-chain amino acids, 5-oxoproline, ophthalmate, and the ophthalmate: norophthalmate ratio, associated with decreased γ-glutamyl cysteine, glutamate, and norophthalmate. Lipid disturbances were evident from changes in lysophospholipids and lysoplasmalogens and increased PUFAs. There were also changes in purine and pyrimidine nucleosides and nucleotides. There is abundant evidence of increased release of ROS and peroxidation of membrane phospholipids and generation of reactive aldehydes in AD, from the early stages of the disease (Section 5.1.3). The best indicators of peroxidation [369] include F_2_-isoprostanes, which arise from arachidonic acid [370], and F_4_-isoprostanes (neuroprostanes) derived from eicosapentaenoic acids or docosahexaenoic acids which contribute to neurodegenerative diseases [371]. Other reliable markers of oxidative stress are acrolein and its conjugates; 7-ketocholesterol (7KC), a toxic product of a reaction between cholesterol and ROS [372,373]; and 8-oxoguanine (8-oxoG) and its nucleotide 8-oxo-2’-deoxyguanosine (8-oxodG), the guanine and deoxyguanosine oxidation products, respectively, regarded as the most significant biomarkers for oxidative DNA damage [374,375].

Some of the compounds listed here might be useful markers.

### 8.2. Suggestions for Further Study

Investigate neurons and astrocytes together, possibly in organ cultures, in view of their tight symbiosis.

Clarify the roles of ApoE4.

Consider why ApoE4 increased arterial vasospasm after subarachnoid haemorrhage (SAH) and the beneficial action of an ApoE4-mimetic peptide.

Elucidate the roles of mitochondrial-derived peptides in regulating ATP synthesis.

Clarify the roles of APP/Aβ in the endoplasmic reticulum.

Cross-link with cardiologists investigating memory and cognitive impairment following cardiac events and neurosurgeons following SAH.

Consider the other BACE substrates and whether any may generate damaging fragments in AD.

Investigate the effects of SLC25A25 knockdown on the metabolome and proteome of brain cells to observe the impact of ATP depletion [84,85].

Consider the administration of agents to promote the anaplerotic capacity of astrocytes to bolster ATP production.

## 9. Conclusions

The identity of the factor(s) which initiates the brain damage that leads to Alzheimer’s disease remains elusive. The proposal that poor perfusion with reduced oxygen delivery to the hippocampus triggers the process through ATP depletion is attractive. Certainly it would contribute to ongoing damage and open new avenues for therapy and early detection of AD. There is good evidence to implicate hypoperfusion. There is a strong scientific argument that ATP depletion could be a major factor in initiating and propagating the pathological disturbances that characterize AD, and there is evidence from a variety of sources to support this.

## Figures and Tables

**Table 1:** Risk factors for Alzheimer’s disease (AD).

Factor
Age
Overweight in midlife, but not late-life overweight or obesity [10]
High blood cholesterol; familial hypercholesterolaemia [10]
Low HDL cholesterol [10]
Persistent hypertension-midlife [10]
Smoking [11]
Chronic stress [11]
Depression [11]
Alcohol-heavy intake or abstinence [12]
Poor sleep [11]
Recurrent hypoxia-sleep apnoea, Chronic obstructive pulmonary disease COPD [13]
Hyperammonaemia (potential risk) [14,15]
Genes and polymorphisms
*APOE4* [16]
*TREM2* Triggering Receptor Expressed on Myeloid cells 2 [11]
*MT-RNR2* Humanin, Mitochondrially encoded 16S RNA rs2854128—African Americans (not Europeans) [17]
*SLC25A22* Solute Carrier Family 25 Member 22 (Glutamate/H(+) Symporter 1) [18]
*ABCA1* ATP-binding cassette subfamily A member 1 loss of function [19]
*ABCA7* ATP-binding cassette subfamily A member 7 numerous polymorphisms [20,21]
*SREBP-2* Sterol Regulatory Element Binding Transcription Factor 2 [22]
*MBOAT1* Membrane-Bound Glycerophospholipid O-Acyltransferase 1 (proposed risk) [23]
*PICALM* phosphatidylinositol binding clathrin assembly protein rs3851179 [24]
Mutations in genes for amyloid precursor protein (APP), presenilin 1 (PSEN1), and presenilin 2 (PSEN2) rare early-onset AD [25]

**Table 2:** Sensors of ATP depletion and their actions to restore ATP levels.

Signal of Low ATP	Sensor/Mediator	Actions to Increase ATP Supply	Actions to Decrease ATP Consumption
Raised AMP/ATP or ADP/ATP ratio	AMP-activated protein kinase (AMPK) [52,57,58,59]	↑ Glucose transporters 1 and 4 (GLUT 1/4)	↓ Fatty acid synthesis
		↑ Glycolysis	↓ Cholesterol synthesis
		↑ Fatty acid uptake by mitochondria	↓ Triacylglycerol phosphate synthesis
		↑ Mitochondrial biogenesis	↓ Transcription of lipogenic enzymes
		↑ Mitochondrial fission	↓ Glycogen synthesis
		↑ Mitochondrial ADP/ATP translocase (ANT) activity	↓ rRNA synthesis
		↑Autophagy and mitophagy	↓ Protein synthesis
		↑NAD^+^/NADH ratio and inhibits Sirtuin 1	
Raised AMP/ATP or ADP/ATP ratio? via AMPK activation	Mitochondrial ADP/ATP translocase [73,74,75,76]	Increased mitochondrial ADP uptake and ATP release	
Low NAD^+^/NADH ratio in hypoxia	Sirtuin 1 (SIRT1) (down-regulated) [66,67]	With down-regulation, ↑ acetylation and activation of HIF1α	
Raised AMP/ATP ratio + Ca^2+^ release through stress hormone stimulation	Mitochondrial Ca^2+^-regulated ATP-Mg^2+^/phosphate carrier SLC25A25 [73]	↑ ATP-Mg^2+^ release from mitochondria	
Oxygen depletion (hypoxia)	HIF-1α † [86,87,88,89]	↑Erythropoietin stimulates red blood cell production, ↑VEGF (vascular endothelial growth factor) increases angiogenesis and increases O_2_ delivery. ↑ Glucose transporters 1 and 3 (GLUT 1/3)	Increases conversion of extracellular ATP to adenosine which inhibits the NMDA receptor and glutamate signalling [111]
Oxygen depletion (hypoxia)	HummR [86,89]	↑ axonal mitochondrial transport	
Uncertain at present	Mitochondrial-derived peptides/nuclear-encoded mitochondrial peptides [17]		
↑ AMP	Phosphofructokinase [57,58]	↑ Glucose catabolism	
↑ AMP	Glycogen phosphorylase [57,58]	↑ Glucose release from glycogen	
↑AMP	Fructose 1,6-bisphosphatase (inhibited) [57,58]		↓ gluconeogenesis

HIF-1α † activates numerous genes. The main targets are erythropoietin and vascular endothelial growth factor (VEGF); ↑ increased activity; ↓ decreased activity.

**Table 3:** Investigations of the hippocampal vasculature and blood flow.

	Study	Main Findings	Reference
	Human studies		
1	Atherosclerosis of Circle of Willis arteries in AD	Number of stenoses and stenosis index in AD > controls; correlated with plaque, NFTs, white matter rarefaction, Braak stage	Roher AE, Esh C, Kokjohn T, et al., 2003 [281]
2	Atherosclerosis of cerebral arteries in AD	Stenosis of arteries and number of stenoses per individual in AD > controls—highly significant	Roher AE, Esh C, Rahman A, et al., 2004 [282]
3	Vascular hippocampal plasticity after aerobic exercise in older adults	Fitness improvement correlated with changes in hippocampal perfusion and head volume, but considerable interindividual variability in the response to physical exercise	Maass A, Düzel S, Goerke M, et al., 2005 [283]
4	Hippocampal vascularization patterns in vivo	Variable contribution of the anterior choroidal artery, the relationships between hippocampal and posterior cerebral artery patterns, and different distribution patterns in the right and left hemispheres.	Spallazzi M, Dobisch L, Becke A, et al., 2019 [272]
5	Cerebral Angioarchitectonics in AD, compared with other neurodegenerative and ischaemic lesions	temporal and fronto-parietal areas of all patients with AD, regardless of disease stage: specific changes in cerebral microcirculation which they named dyscirculatory angiopathy of Alzheimer’s type (DAAT). DAAT was not found in the controls.	Maksimovich IV, 2018 [284]
6	Effects of acute hypoxia on cerebral bioenergetics and memory.	In hypoxia, oxygen delivery was reduced in middle cerebral artery during central executive tasks and in posterior cerebral artery during memorization and recall; no effect on cerebral blood flow	Ando S, Tsukamoto H, Stacey BS, et al., 2023 [274]
7	Regional cerebral microvascular perfusion in acute and prolonged hypoxia	2 h of hypoxia: perfusion increased in the frontal cortex and decreased in ‘default mode’ network; after 10 h, decreased blood flow in default mode network was more pronounced and widespread, hence reduced local perfusion; showed a relationship with vasoconstriction	Lawley JS, Macdonald JH, Oliver SJ, 2017 [285]
8	Effects of brain ischaemia on succinate and other metabolites	warm ischaemia ex vivo: time-dependent accumulation of succinate; other significant changes included increases in purine degradation, PUFAs, and 5-oxoproline and decreases in adenosine and acylcarnitines; Stroke model: succinate accumulated, other TCA metabolites decreased, and a dramatic decrease in ATP	Mottahedin A, Prag HA, Dannhorn A, et al., 2023 [286]
9	Association of regional cerebral perfusion in AD with Tau and amyloid	Tau-PET was associated with lower CBF in the entorhinal cortex, persisted after excluding AD dementia group, and was independent of Aβ, APOE genotype and MRI markers for small vessel disease. Amyloid-PET was associated with lower CBF in temporo-parietal regions	Rubinski A, Tosun D, Franzmeier N, et al., 2021 [260]
10	Tau deposition in entorhinal cortex related to hypoperfusion	baseline CBF was associated with tau deposition at the 6-year follow-up in the left but not the right entorhinal cortex; findings suggest that a reduction in CBF at the entorhinal cortex precedes tau deposition.	Kapadia A, Billimoria K, Desai P, et al., 2023 [4]
11	Longitudinal changes in CBF in the older hypertensive brain	Relative to controls, in the hypertensive group, rCBF decreased in prefrontal, anterior cingulate, and occipital areas over time	Beason-Held LL, Moghekar A, Zonderman AB, et al. 2007 [287]
	Animal studies		
12	Neurovascular coupling in the hippocampus and visual cortex	Compared with visual cortex, hippocampal arteries had a blunted response: fewer, smaller dilations. ATP production is restricted in tissues furthest from capillaries	Shaw K, Bell L, Boyd K, et al., 2021 [273]
13	Identification of leukotrienes C4 and D4 in gerbil brains after ischaemia and reperfusion.	Significant increases at 5, 10, or 15 min of ischaemia, more marked on reperfusion; highest in forebrain grey matter, undetectable in brain regions remote from ischaemic zone	Moskowitz MA, Kiwak KJ, Hekimian K, et al., 1984 [288]
14	Biochemical response to hypobaric oxygen: hippocampus, cortex, cerebellum	Compared with controls, increased lactate dehydrogenase, free radical generation, lipid peroxidation, glutamate dehydrogenase activity, and vesicular glutamate transporter expression decreased glutathione reductase, superoxide dismutase activity, reduced glutathione with increased oxidized glutathione	Hota SK, Barhwal K, Singh SB, et al., 2007 [289]
15	Hippocampal morphology following hypobaric hypoxia	Significant cell degeneration and death only in the CA3 region; damage is more noticeable with longer time following exposure	Shukitt-Hale B, Kadar T, Marlowe BE, et al., 1996 [290]
16	Oxidative stress in rat brain in hypobaric hypoxia	Significant increase in free radical production, nitric oxide, lipid peroxidation, and lactate dehydrogenase greater at 7 days than 3 days; reduced glutathione, glutathione peroxidase, glutathione reductase, and superoxide dismutase, and reduced/oxidized glutathione. Hippocampus is most susceptible.	Maiti P, Singh SB, Sharma AK, et al., 2006 [291]
17	Effect of acute hypobaric hypoxia on SOD and MDA, and mRNA expression of VEGF and HIF1-α in rat brain	Increased expression of HIF1-α and VEGF on days 1, 2, 3; significant increase in MDA, decreased SOD	Tahir MS, Almezgagi M, Zhang Y, et al. 2021 [292]

NFTs neurofibrillary tangles, CBF cerebral blood flow, rCBF regional cerebral blood flow, PUFAs polyunsaturated fatty acids, *HIF1α*, *Hypoxia-inducible factor 1-alpha*, *VEGF vascular endothelial growth factor*, MDA malondialdehyde, SOD superoxide dismutase.

**Table 4:** Genomic, proteomic, metabolomic, and imaging investigations of human brain and CSF in Alzheimer’s Disease (AD) Studies ^†^.

	Study	Main Findings	Reference
	Brain		
1	Neuronal loss of entorhinal cortex	Controls: neuronal numbers constant 60y to 90y AD: severe neuronal loss; mainly layers II and IV; loss correlated with NFTs and neuritic but not diffuse or total plaques	Gómez-Isla T, Price JL, McKeel DW Jr, et al., 1996 [1]
2	Non-targeted metabolomics to identify pathways altered in AD	Most affected pathway: Ala, Asp, Gln, Asp significant decrease, marked disturbances of malate-aspartate shuttle, glycerophospholipids, pyrimidines; increased S-adenosyl methionine, S-adenosylhomocysteine	Paglia G, Stocchero M, Cacciatore S, et al., 2016 [306]
3	Brain energy pathways in cingulate cortex of young adult *APOE4* carriers without AD	Carriers: increased expression of subunits of mitochondrial complexes I, II, IV; no change in III or V; qPCR: significant small changes in *NDUFB5*, *NDUF7*, and *ARRDC3* expression	Perkins M, Wolf AB, Chavira B, et al., 2016 [16]
4	Brain structural changes over 12–24 m in MRI scans	Mean annualized hippocampal volume change: AD 4.8%, controls 1.1%; AD increased neuronal loss	Ledig C, Schuh A, Guerrero R, et al., 2018 [38]
5	Investigation of gene pathways enriched in hippocampus in AD	In AD, significant changes in NF-κβ, and cGMP-PKG signalling pathways, *MT1*, *MT2*, *NOTCH2*, *ADD3*, *MSX1*, *RAB31,* key hub genes	Liang JW, Fang ZY, Huang Y, et al., 2018 [307]
6	Gene pathway analysis to find biomarkers of human brain ageing	Modules relevant to brain ageing: synaptic vesicle cycle, cGMP-PKG signalling pathway, and oxidative phosphorylation	Hu Y, Pan J, Xin Y, et al., 2018 [47]
7	Changes in proteome and phosphoproteome in AD progression (7)	Identified three proteome clusters associated with AD progression. Enriched pathways were mitochondria, mitochondrial function, and neurotrophic factor signalling	Bai B, Wang X, Li Y, et al., 2020 [308]
8	Gene pathway analysis to identify new gene and miRNA biomarkers for AD	Identified 8 genes, one of which, MBOAT1, was not previously reported, and five *miRNAs*	Soleimani Zakeri NS, Pashazadeh S, MotieGhader H, 2020 [23]
9	Genomic and transcriptomic analyses of hippocampus	Expression of 54 genes associated with AD; 21 were prioritized, including two novel genes *Tyrosine-Protein Phosphatase Non-Receptor Type 9 (PTPN9)* and *Protocadherin Alpha 4 (PCDHA4)*; *QPCTL (glutamyl cyclotransferase*) and *ERCC2 (excision repair 2)* were significantly different from elderly controls	Liu N, Xu J, Liu H, et al., 2021 [309]
10	Investigation of co-expression networks and regulators of metabolism in AD progression	With AD progression, decreased branched-chain AAs, and short-chain acylcarnitines, increased medium- and long-chain acylcarnitines, increased expression of adiponectin protein and *ATP-Binding Cassette Subfamily A Member 1 (ABCA1)* and *Carnitine Palmitoyltransferase 1A* (*CPT1A*) genes in the Hippocampus and para-hippocampal gyrus	Horgusluoglu E, Neff R, Song W-M, et al., 2021 [310]
11	Identification of gene pathways in brain regions with AD pathology identified by use of three different PET scans	Results from Tau scans are most relevant. Pathways identified included mitochondrial respiration, electron transport, OXPHOS, and metabolism	Mullins R and Kapogiannis D, 2022 [311]
12	Transcriptomic analyses of hippocampal entorhinal subfields to identify regulators in AD	All 5 subfields were positively enriched in AD signalling pathways, extensive neuronal loss in all 5 regardless of AD pathology; most differentially expressed genes in EC and CA4, with a significant correlation of neuronal and astrocyte profiles, PSP (prosaposin), a key modulator of astrogliosis	Luo D, Li J, Liu H, et al., 2023 [312]
13	Changes in brain protein expression with AD progression to find proteins to predict progression of MCI to AD, using machine learning	29 proteins provided best classification of AD and controls; 88 proteins were needed to classify AD and asymptomatic AD; predictive proteins of change with disease state were significantly enriched for sugar metabolism supporting dysregulation of energy metabolism	Tandon R, Levey AI, Lah JJ, et al., 2023 [313]
14	Association of 53 SLC25 carriers with AD	*SLC25A10*, *SLC25A17*, and *SLC25A22* were identified as AD susceptibility genes; down-regulation of gene for glutamate carrier 1 (*SLC25A22)* is associated with accelerated hippocampal atrophy and increased hazard of dementia. Pathway analysis related *SLC25A22* to defects in neuronal function	Tian J, Jia K, Wang T, et al., 2024 [18]
15	Human cortical peptidome in cognitive resilience against AD	35 proteins were significantly associated with resilient AD (AD pathology but normal cognition) or with low cognition without AD pathology. In resilient increased ATP synthase F1 subunit delta (ATP5FLD) and cytochrome C oxidase subunit 8A (COX8A) were found. Heterogeneous Nuclear Ribonucleoprotein K (HNRNAP) was enriched in inhibitory neurons	Morgan GR and Carlyle BC, 2024 [2]
16	Comprehensive hippocampal bio-informatics study using machine learning to identify novel risk genes for AD	27 down-regulated and 4 up-regulated genes correlated with AD stage. Higher expression of five genes was associated with decreased risk and slower progression of AD; four with higher risk and faster progression: *PNMAL1*, *SLC39A10*, *GLRB*, and *PTPN3*	Li J, Li L, Cai S, et al., 2024 [11]
	CSF		
17	CSF Metabolite profiles in AD	In mild AD, compared with controls, combination of significantly increased cysteine and decreased uridine is 75% predictive of AD, with sensitivity of 75%; Cortisol increased with progression of AD in more advanced AD, i.e., increased cortisol	Czech C, Berndt P, Busch K, et al., 2012 [314]
18	Untargeted CSF metabolomics in prodromal AD with mild cognitive impairment	94 of 294 differentially expressed metabolites were annotated; disturbance in 13 pathways was identified. Top four pathways related to bioenergetics and glucose metabolism (N-glycan, sialic acid, amino sugars, galactose); methionine, tyrosine, purine, and biopterin metabolism were also differentially activated	Hajjar I, Liu C, Jones DP et al., 2020 [315]
19	Unbiased CSF proteomics in patients with AD	Compared to non-AD groups, pyruvate kinase (PKM) and aldolase A (ALDOA) were up-regulated in AD CSF, glucose increased only in MCI; 33 peptides were differentially abundant between AD with dementia and all non-demented-AD groups, including clusters for glycolytic processes or canonical glycolysis and synaptic and immune response markers	de Geus MB, Leslie SN, Lam T, et al., 2023 [316]
20	CSF Proteome and metabolome of individuals with varying amyloid/taurine (AT) pathology and nine biomarkers of neurodegeneration and neuroinflammation	61 proteins were significantly associated with AT category, and 636 proteins with biomarkers. Glucose and carbon metabolism pathways were enriched among amyloid- and tau-associated proteins, including malate dehydrogenase, aldolase A, and succinyl carnitine. Preliminary findings supported association of glucose metabolic dysregulation with alterations in amyloid and tau even before cognitive impairment; preliminary investigations suggested possible abnormalities in insulin signalling	Panyard DJ, McKetney J, Deming YK, et al., 2023 [317]

^†^ For more information, refer to Supplementary Table S2. *NDUFB5, NADH: Ubiquinone Oxidoreductase Subunit A5, NDUF7, NADH: Ubiquinone Oxidoreductase Subunit, ARRDC3, Arrestin Domain Containing 3, MT1, A metallothionein 1 gene, MT2, A metallothionein 2 gene, NOTCH2, Notch Receptor 2, ADD3, Adducin 3, MSX1, Msh Homeobox 1, RAB31*, Member RAS Oncogene Family, *SLC25A10,17,22: Solute Carrier Family 25: A10 Mitochondrial Dicarboxylate Carrier, A17 Peroxisomal Membrane Protein 34kD. A17 Glutamate/H (+) Symporter 1, PNMAL1, PNMA8A (Paraneoplastic antigen-like protein 8A, SLC39A10, Solute Carrier Family 39 Member 10* (Zinc-influx transporter), *GLRB, Glycine Receptor Beta PTPN3 Protein Tyrosine Phosphatase Non-Receptor Type 3*, cGMP-PKG, cGMP-protein kinase G.

**Table 5:** Metabolomic, proteomic, and genomic studies of AD in animals ^†^.

	Study	Main Relevant Findings	Reference
21	CSF metabolome of a rabbit model for late-onset AD with AD neuropathology induced by a high-cholesterol diet	Profiles changed with time; Aβ-like plaques were only seen at 12 weeks. Four clusters were identified in the top 95 metabolites, most at 12 weeks. At 12 weeks, decreased phospholipids, mainly phosphorylated fatty alcohols, akylacyl, or dialkyl-glycerophosphates, all potential precursors or degradation products of phospholipids including phosphatidylcholines and plasmalogens.	Liu QY, Bingham EJ, Twine SM, et al., 2012 [26]
22	Cerebral cortical and glutamine metabolism in a mouse AD model (APP_swe_/PSEN1dE9)	AD mice: significantly increased lactate and alanine, decreased TCA intermediates, decreased capacity for uptake and oxidative metabolism of glutamine; no change in glial acetate metabolism.	Andersen JV, Christensen SK, Aldana BI, et al., 2017 [318]
23	Hippocampal proteomic pathways associated with memory status in normal ageing and 5FXAD AD mouse model	Normal and AD mice, HDAC4 identified as regulator of memory-related proteins; top pathways associated with memory deficits in controls: OXPHOS, mitochondrial dysfunction, glutamate receptor signalling.	Neuner SM, Wilmott LA, Hoffmann BR, et al., 2017 [319]
24	Investigation for overlap in protein expression up to 15 m of normal mice following mild traumatic brain injury (TBI) aged 3 m; and non-traumatized mice with AD (PSAPP and mice expressing hTau) up to 15 m	Impaired in TBI: energy metabolism, clearance, neurotransmitter and intracellular signalling, and glial cell function. Little overlap with altered proteins in AD models. TBI and AD damage distinct processes	Ojo JO, Crynen G, Algamal M, et al., 2020 [320]
25	Characterization of Tg4-42 mouse model for AD [321]	Significant loss of hippocampal CA1 neurons. At 9 m caudate, putamen: significant decreases: GABA, glutamine, lactate: increased Aβ42, glutaminase, glutamine decarboxylase, CSF, increased neurofilament light chains (NFL)	Hinteregger B, Loeffler T, Flunkert S, et al., 2021 [321]
26	Metabolite analyses of cortex and hippocampus of a transgenic AD mouse model with high-resolution magic angle spinning NMR.	Controls: changes with age in cortex; at 9 m sex differences; at 9 m differences from AD mice in hippocampus: glutamate, glutamine, Nacetylaspartate (NAA), glycine, phosphocholine, and glycerophosphocholine.	Füzesi MV, Muti IH, Berker Y, et al. 2022 [322]
27	Investigation of mitochondrial dysfunction and effects of an antibody to a neurotoxic Tau peptide in hippocampus and retina of a mouse AD model	Decreased expression of genes involved in multiple energy-generating mitochondrial pathways including OXPHOS pathways; FA oxidation in the hippocampus and retina of Tg2576 AD mice; GSEA analysis: oxidative phosphorylation is the most down-regulated gene set in hippocampus of early symptomatic Tg2576; mitochondrial alterations were observed in AD mice significantly reverted by NH2htau antibody.	Morello G, Guarnaccia M, La Cognata V, et al., 2023 [323]

^†^ For more information, refer to Supplementary Table S3. HDAC4 Histone Deacetylase 4; GSEA, gene set enrichment analysis; Tg25476 AD mice, which overexpress a mutated form of APP (the ‘Swedish mutation’); APP_swe_/PSEN1dE9 (PSAPP) AD mice, which carry the Swedish mutation and a presenilin mutation; 5xFAD mice, which express 5 mutations in two genes (APP and Presenilin-1); and Tg4-42 mice, which carry N-truncated 4-42 Aβ.

## Data Availability

No new data were created. Supplementary information to the data presented in the text is provided in Supplementary Tables S1–S3, with references to the studies reported.

## Supplementary Materials

The following supporting information can be downloaded at https://www.mdpi.com/article/10.3390/ijms26157328/s1.

## Abbreviations

AMPK5′	AMP-activated protein kinase
APH1 (Anterior Pharynx defective 1)	a component of the gamma-secretase complex
APP	Amyloid Beta Precursor Protein
BACE	Beta-Secretase APP Beta-Secretase
BBB	blood–brain barrier
CBF	cerebral blood flow
rCBF	regional cerebral blood flow
FDG-PET	fluorodeoxyglucose (FDG)-positive emission tomography (PET)
GSEA	gene set enrichment analysis
HDAC4	Histone Deacetylase
HIF1α,	Hypoxia-inducible factor 1-alpha
HUMMR	hypoxia up-regulated mitochondrial movement regulator
LOAD	late-onset Alzheimer’s disease
MCI	Mild Cognitive Impairment
Miro1 and Miro2	Mitochondrial Rho GTPase proteins
Mitochondrial-derived peptides:	
*GAU*	*gene antisense ubiquitous*
*MOTS-c*	*Mitochondrial ORF of the 12S rRNA Type-C*
*MtALTND4*	protein encoded from an alternative open reading frame of the gene for the *NADH-ubiquinone oxidoreductase chain 4 (ND4) protein*
*SHLP1 -SHLP6*	six small humanin-like peptides with 20–35 amino acids
*SHMOOSE*	*Small Human Mitochondrial ORF Over SErine tRNA*
MRI	Magnetic Resonance Imaging
NFTs	neurofibrillary tangles,
PEN2,	Gamma-secretase subunit
PET	positive emission tomography
PS1/PS2	Presenilin 1/2
PUFAs	polyunsaturated fatty acids
SAH	subarachnoid haemorrhage
*SREBP-2*	Sterol regulatory element binding protein-2
Transgenic mouse models:	
Tg25476 AD mice	overexpress a mutated form of APP (the ‘Swedish mutation’). Develop amyloid plaques and cognitive deficits
APP_swe_/PSEN1dE9 (PSAPP)	carry both the Swedish mutation and a presenilin mutation
5xFAD mice	express 5 mutations in two genes *(APP* and *Presenilin-1*); have increased Aβpeptide, amyloid plaques, and cognitive deficits
3xTG mice	express the Swedish mutation, and mutations in PSEN1and human Tau
Tg4-42 mouse model	express N-truncated 4-42 Aβ
