# Supplementary material for: Are Hippocampal Hypoperfusion and ATP Depletion Prime Movers in the Genesis of Alzheimer’s Disease? A Review of Recent Pertinent Observations from Molecular Biology
Source: Int J Mol Sci. 2025 Jul 29;26(15):7328. doi: 10.3390/ijms26157328 (PMC12347683; doi:10.3390/ijms26157328)
Supplement: Supplementary file 1 [file ijms-26-07328-s001.zip › Suppl table 2 VW Human Brain studies in AD.pdf]

+

Supplementary Table S2. Proteomic and Metabolomic Investigations of Human Brain and CSF in Alzheimer's Disease (AD) Studies

Study		Tissue	Methods	Main findings
	**Brain**			
1	Neuronal loss of entorhinal cortex [1]	Entorhinal cortex from controls and AD patients with graded cognitive impairment	Neuronal counts, NFTs & amyloid plaques on brain sections	Controls: neuronal numbers constant 60y to 90y AD: severe neuronal loss; mainly layers II and IV; loss correlated with NFTs and neuritic but not diffuse or total plaques
2	Non-targeted metabolomics to identify pathways altered in AD [308]	Frontal cortex from controls and AD patients < 4h after death	Untargeted lipidomics, metabolomics and mass spectrometric imaging of brain sections;	Most affected pathway: Ala, Asp, Gln, Asp significant decrease, marked disturbances of malate-aspartate shuttle, glycerophospholipids, pyrimidines; increased S-adenosyl methionine, S-adenosylhomocysteione
3	Brain energy pathways in cingulate cortex of young adult ApoE4 carriers without AD [16]	Brain cingulate cortex from young ApoE4 carriers & non-carriers compared	Quantitative histochemistry of OXPHOS protein subunits; mitochondrial energy q PCR array	Carriers: increased expression of subunits of mitochondrial complexes I,II, IV, no change in III or V; qPCR: significant small changes in *NDUFB5, NDUF7, ARRDC3* expression
4	Brain structural changes over 12-24m in MRI scans [38]	Healthy controls & AD patients from ADNI Neuroimaging cohort	MRI imaging	Mean annualized hippocampal volume change AD 4.8%, controls 1.1%; AD increased neuronal loss
5	Investigation of gene pathways enriched in hippocampus in AD [309]	Gene expression data in hippocampus: patients with AD of graded severity (22), controls (9) from an AD study cohort	Weighted Gene Co-expression Analysis (WGNCA), GO and KEGG pathway analysis. qRT-PCR of hub genes in AD transgenic mice	In AD, significant changes in NF-κβ, and cGMP-PKG signalling pathways, MT1, MT2, NOTCH2, ADD3, MSX1, RAB31 key hub genes
6	Gene pathway analysis to find biomarkers of human brain aging [47]	GSE53890 public microarray gene data set. Brain sites not indicated	WGCNA, GO enrichment and KEGG pathway analysis ; validated with independent brain aging data sets	Modules relevant to brain aging: synaptic vesicle cycle, cGMP-PKG signalling pathway, and oxidative phosphorylation
7	Changes in proteome & phosphoproteome in AD progression [310]	Frontal cortex of brain from controls and patients with AD of graded severity from two independent cohorts; mouse 5xAD model for validation	Analyses: multiplexed and 2D liquid chromatography-tandem MS; interpretation on-line protein-protein interaction network	Identified three proteome clusters associated with AD progression. Enriched pathways were mitochondria, mitochondrial function, neurotrophic factor signalling

8	Gene pathway analysis to identify new gene and miRNA biomarkers for AD [23]	AD gene data set from National Center for Biotechnology Information Gene expression Omnibus (GEO), including AD (145), MCI (80), controls (104)	WGNCA, identification of relevant gene modules, then functional and enrichment analysis of selected genes and mi RNA	Identified 8 genes, *ARMC7*, *HNRNPUL1*, *LAMTOR1, PLAGL2, CREBRF, LCOR, MRI1, MBOAT1* (not previously reported) and five miRNAs
9	Genomic and transcriptomic analyses of hippocampus [311]	Genomic and transcriptomic data of 111 hippocampal samples and data from two large GWAS studies, replicated in a third cohort	Transcriptome-wide analysis, integrated hippocampal gene expression and GWAS data	Expression of 54 genes associated with AD; 21 were prioritised, including two novel genes *Tyrosine-Protein Phosphatase Non-Receptor Type 9 (PTPN9)* and *Protocadherin Alpha 4 (PCDHA4)* ; *QPCTL (glutamyl cyclotransferase,* and *ERCC2 (excision repair 2)* significantly different from elderly controls
10	Investigation of co-expression networks and regulators of metabolism in AD progression [312]	Brain transcription data for controls & patients with AD from three centres, fasting blood for metabolite, transcriptome and proteome analyses and clinical data from two of the centres	Constructed a co-expression network for metabolites, prioritised co-expressed modules based on cognition and brain pathology, compared with brain gene transcription sets to assess relevance.	With AD progression, decreased branched chain AAs, and short chain acylcarnitines, increased medium and long chain acyl carnitines, increased expression of adiponectin protein and *ATP-Binding Cassette Sub-Family A Member 1 (ABCA1)* and *Carnitine Palmitoyltransferase 1A* (*CPT1A*) genes in the Hippocampus and para hippocampal gyrus
11	Identification of gene pathways in brain regions with AD pathology identified by use of three different PET scans [313]	PET scan images of AD and CN participants in the ADNI data base;<br>For correlation: cerebral cortex gene microarrays from autopsy samples from 6 cognitively normal subjects	PET scans: FDG-18 (glucose uptake), AV-45 (Aβ amyloid), AV-1451(Tau). Correlated with gene expression data in affected areas	Results from Tau scans most relevant. Pathways identified included mitochondrial respiration, electron transport, OXPHOS and metabolism
12	Transcriptomic analyses of hippocampal entorhinal subfields to identify regulators in AD [314]	Post-mortem brain from controls and patients with AD (Chinese) Hippocampal sections from CA1 to CA4 and entorhinal cortex (EC)	WGCNA analysis of transcriptome data set & GO & KEGG analyses of modules to identify enriched pathways	All 5 subfields positively enriched in AD signalling pathways, extensive neuronal loss in all 5 regardless of AD pathology; most differentially expressed genes in EC and CA4, significant correlation of neuronal and astrocyte profiles, PSP (prosaposin) a key modulator of astrogliosis
13	Changes in brain protein expression with AD progression to find proteins to predict progression of MCI to AD, using machine learning [315]	Brain proteomic analyses of controls and AD patients from 6 AD cohorts, 4 discovery, 2 validation, samples dorsolateral prefrontal cortex (5), temporal cortex (1)	Label-free quantification and machine learning	29 proteins provided best classification of AD and controls; 88 proteins needed to classify AD and asymptomatic AD; predictive proteins of change with disease state were significantly enriched for sugar metabolism supporting dysregulation of energy metabolism
14	Association of 53 SLC25 carriers with AD [18]	Whole -genome sequencing GWAS data from two large	Transcriptome-wide association of SLC25 genes	*SLC25A10*, *SLC25A17*, and *SLC25A22* identified as AD susceptibility genes, down regulation of gene

		discovery cohorts of patients with AD & controls, & GWAS & MRI data from the ADNI cohort for validation.	with AD; Referred to Consortium atlas of genetic regulatory effects to identify genes likely to be expressed in hippocampus	for glutamate carrier1 (*SLC25A22*) associated with accelerated hippocampal atrophy and increased hazard of dementia. Pathway analysis related SLC25A22 to defects in neuronal function
15	Human cortical peptidome in cognitive resilience against AD [2]	Postmortem brain cortical angular gyrus from AD patients grouped according to cognition & AD pathology	LC-MS analyses; published single-nucleus RNA Seq data to link differentially enriched peptides to cell types	35 proteins were significantly associated with resilient AD (AD pathology but normal cognition) or with low cognition without AD pathology. In resilient, increased ATP synthase F1 subunit delta (ATP5FLD), cytochrome C oxidase subunit 8A (COX8A). Heterogeneous Nuclear Ribonucleoprotein K (HNRNAP) was enriched in inhibitory neurons
16	Comprehensive hippocampal bio-informatics study using machine learning to identify novel risk genes for AD [11]	RNA Seq hippocampal data sets from Gene Expression Omnibus, AD plus controls	Stratified AD sample data using 9 genes for AD features and three distinct machine learning methods.	27 down-regulated and 4 up-regulated genes correlated with AD stage. Higher expression of *YWHAZ, PITHD1, SCG5, YWHAH, TUBB* genes associated with decreased risk & and slower progression of AD; 4 with higher risk & faster progression *PNMAL1, SLC39A10, GLRB, PTPN3*
	**CSF**			
17	CSF Metabolite profiles in AD [316]	CSF from controls (51) and patients with AD aged ≥ 40y (79) from 5 European centres; MRI within preceding 12m	Metabolites analysed with GC-MS and LC-MS/MS, Tau and Aβ immunochemically	In mild AD, compared with controls: combination of significantly increased cysteine and decreased uridine 75% predictive of AD, with sensitivity of 75%; Cortisol increased with progression of AD in more advanced AD increased cortisol
18	Untargeted CSF metabolomics in prodromal AD with mild cognitive impairment [317]	Fasting CSF from individuals ≥ 50y with normal cognition or mild cognitive impairment (MCI)	Metabolites analysed with high resolution LC-MS and for pathway enrichment	94 of 294 differentially expressed metabolites were annotated; disturbance in 13 pathways identified. Top four pathways related to bioenergetics and glucose metabolism (N-glycan, sialic acid, amino sugars, galactose); methionine, tyrosine, purine and biopterin metabolism also differentially activated
19	Unbiased CSF proteomics in patients with AD [318]	AD patients with MCI or with dementia (DEM-AD) or cognitively unimpaired (CU) attending a neurological clinic, aged 56-94y.	Deep proteomic analysis using unbiased mass spectrometry	Compared to non-AD groups pyruvate kinase (PKM) and aldolase A (ALDOA) upregulated in AD CSF, glucose increased only in MCI; 33 peptides were differentially abundant between AD with dementia and all nondemented-AD groups, including clusters for glycolytic process or canonical glycolysis, synaptic and immune response markers

20	CSF Proteome and metabolome of individuals with varying amyloid/taurine (AT) pathology and nine biomarkers of neurodegeneration and neuroinflammation [319]	Two cohorts, cognitively unimpaired 74.5%, MCI 11.7%; AD dementia 13.9% Replicated in a third cohort	Ultrahigh performance LC-tandem MS; subset of samples analysed for proteins in insulin signalling pathways. Findings were related to 9 recognised CSF AD biomarkers	61 proteins significantly associated with AT category and 636 proteins with biomarkers. Among amyloid- and tau-associated proteins proteins from glucose and carbon metabolism pathways were enriched, including malate dehydrogenase, aldolase A and succinyl carnitine; Preliminary findings supported association of glucose metabolic dysregulation with alterations in amyloid and tau even before cognitive impairment; preliminary investigations suggested possible abnormalities in insulin signalling
