# Supplementary material for: Are Hippocampal Hypoperfusion and ATP Depletion Prime Movers in the Genesis of Alzheimer’s Disease? A Review of Recent Pertinent Observations from Molecular Biology
Source: Int J Mol Sci. 2025 Jul 29;26(15):7328. doi: 10.3390/ijms26157328 (PMC12347683; doi:10.3390/ijms26157328)
Supplement: Supplementary file 1 [file ijms-26-07328-s001.zip › Suppl Table 3 VW animal studies relevant to AD.pdf]

Supplementary Table S3 Metabolomic, proteomic and genomic studies of AD in animals

Study		Model	Methods	Main relevant findings
21	CSF metabolome of a rabbit model for late onset AD with AD neuropathology induced by a high cholesterol diet [26]	1.5y old rabbits fed high (2%) cholesterol diet for up to12 weeks and controls; CSF from cisterna magna at 4,8,12 weeks.	Fourier transform ion cyclotron resonance (FTICR) MS. Metabolite expression differing significantly from controls at different time points. Aβ40 and Aβ42 quantified in cortex and hippocampus at 12 weeks	Profiles changed with time; Aβ-like plaques only seen at 12 weeks Four clusters identified in the top 95 metabolites, most at 12 weeks. At 12 weeks, decreased phospholipids, mainly phosphorylated fatty alcohols, akylacyl or dialkyl-glycerophosphates, all potential precursors or degradation products of phospholipids including phosphatidylcholines and plasmalogens
22	Cerebral cortical and glutamine metabolism in a mouse AD model (APP_{swe}/PSEN1dE9) [320]	APP_{swe}/PSEN1dE9 AD mice aged 3m, before amyloid plaque developed, and controls	Acutely isolated cortical & hippocampal slices incubated with [$U^{13}C$] glucose, [1,2 ^{13}Cacetate] or $U^{13}C$ glutamine. Analysed with mass spectrometry	AD mice: significantly increased lactate and alanine, decreased TCA intermediates, decreased capacity for uptake and oxidative metabolism of glutamine; no change in glial acetate metabolism.
23	Hippocampal proteomic pathways associated with memory status in normal aging and 5FXAD AD mouse model [321]	5FXAD AD mice, nontransgenic controls classed as impaired or intact according to memory status (fear test)	Differential expression of proteins in snap frozen hippocampal membranes analysed with LC-MS-MS	Comprehensive analysis of hippocampal proteins in normal & AD mice. HDAC4 identified as regulator of memory-related proteins; Top pathways associated with memory deficits in controls: OXPHOS, mitochondrial dysfunction, glutamate receptor signalling;
24	Investigation for overlap in protein expression up to 15 m of normal mice following mild traumatic brain injury (TBI) aged 3m; and non-traumatised mice with AD († PSAPP and mice expressing hTau) up to 15 m [322]	Wild-type C57BL/6 controls, mice expressing human tau (hTau tauropathy model), and PSAPP mice, expressing the double KM670/671NL (Swedish) and Presenilin 1 M146L mutations (amyloid model)	[i] brain injury study: Wild type mice aged 3m: injury (5 sharp spikes over 9 days) or sham-operated, cortex & hippocampus analysed 24h, 3, 6, 9, or 12 months later<br>[ii] hTau & PSAPP mice-brain cortex analysed aged 3, 9 or 15m<br>Unbiased proteomic analysis with LC-MS/MS	Impaired in TBI: energy metabolism, clearance, neurotransmitter and intracellular signalling, glial cell function. Little overlap with altered proteins in AD models. TBI and AD damage distinct processes
25	Characterization of †Tg4-42 mouse model for AD [323]	Mouse model with express N-truncated human 4- 42 Aβ (not produced physiologically); has	Immunohistochemistry of hippocampus; non-targeted	Significant loss of hippocampal CA1 neurons.

		learning disturbance and hippocampal neuronal loss; intracellular Aβ accumulation in the brain without plaque formation	metabolic profiling (NMRS) of cortex, caudate and putamen GLU/GABA/GLN pathway decreased	At 9m, caudate, putamen: significant decreases: GABA, glutamine, lactate: increased Aβ42, glutaminase, glutamine decarboxylase, CSF, increased neurofilament light chains (NFL)
26	Metabolite analyses of cortex & hippocampus of a transgenic AD mouse model with high resolution magic angle spinning NMR [324]	Transgenic AD mice and wild type controls aged 3m,9m,18m	High resolution magic angle spinning (HRMS) NMR analysis	Controls: changes with age in cortex; at 9m sex differences; at 9m differences from AD mice in hippocampus: glutamate, glutamine, Nacetylaspartate (NAA), glycine, phosphocholine and glycerophosphocholine.
27	Investigation of mitochondrial dysfunction and effects of an antibody to a neurotoxic Tau peptide in hippocampus and retina of a mouse AD model [325]	Hippocampus and retina of Tg25476 AD mice, control animals, and Tg mice treated with a monoclonal antibody to NH2htau peptide, at 6m of age	Gene set enrichment analysis (GSEA) of the transcriptomes for functional interpretation, validation by RT-rPCR of a core set of genes, comparison with independent data sets deposited with GEO hippocampus at 5m and cerebral cortex at 2m,9m and12m of Tg25476 AD mice<br>[of interest: succinic dehydrogenase decreased]<br>From GSEA validation some of the down-regulated genes decreased in cortex at 2m-long before amyloid]	Decreased expression of genes involved in multiple energy generating mitochondrial pathways including OXPHOS pathways; FA oxidation; in the hippocampus and retina of Tg2576 AD mice; gene set enrichment analysis G (SEA) analysis: oxidative phosphorylation the most down-regulated gene set in hippocampus of early symptomatic Tg2576; mitochondrial alterations observed in AD mice significantly reverted by NH2htau antibody.

Transgenic mouse models: Tg25476 AD mice: overexpress a mutated form of APP (the 'Swedish mutation'), APP_{swe}/PSEN1dE9 (PSAPP) AD mice: carry two mutations: the Swedish mutation and a presenilin mutation, 5xFAD mice express 5 mutations in two genes (APP and Presenilin-1), mouse model with N-truncated 4- 42 Aβ

HDAC4 Histone Deacetylase 4, GSEA gene set enrichment analysis
