# Supplementary material for: Are Hippocampal Hypoperfusion and ATP Depletion Prime Movers in the Genesis of Alzheimer’s Disease? A Review of Recent Pertinent Observations from Molecular Biology
Source: Int J Mol Sci. 2025 Jul 29;26(15):7328. doi: 10.3390/ijms26157328 (PMC12347683; doi:10.3390/ijms26157328)
Supplement: Supplementary file 1 [file ijms-26-07328-s001.zip › Suppl Table1 VW Iinvestigations of cerebral blood flow.pdf]

Supplementary Table S1 Investigations of the hippocampal vasculature and blood flow

Study		Study group	Method	Main findings
	**Human studies**			
1	Atherosclerosis of Circle of Willis arteries in AD [282]	Autopsy, sporadic AD (32), non-demented controls (22)	Calculated stenosis index of 3mm arterial cross-sections	Number of stenoses and stenosis index in AD> controls; correlated with plaque, NFTs, white matter rarefaction, Braak stage
2	Atherosclerosis of cerebral arteries in AD [283]	Autopsy, sporadic AD (10), controls (10)	Calculated stenosis index of large leptomeningeal and circle of Willis arteries	Stenosis of arteries and number of stenoses per individual in AD > controls- highly significant
3	Vascular hippocampal plasticity after aerobic exercise in older adults [284]	Healthy older adults (60-77 years) who undertook aerobic exercise for 3 months	Regional cerebral blood flow measured with gadolinium-based perfusion MRI Hippocampal volumes with high-resolution 7 Tesla MRI.	Fitness improvement correlated with changes in hippocampal perfusion and head volume, but considerable interindividual variability in the response to the physical exercise
4	Hippocampal vascularization patterns in vivo [273]	Healthy young adults (41)	High resolution 7-tesla time-of-flight angiography (0.3mm resolution); different vascularization patterns involved in hippocampal blood supply	Variable contribution of the anterior choroidal artery, the relationships between hippocampal and posterior cerebral artery patterns, different distribution patterns of the right and left hemispheres.
5	Cerebral angioarchitectonics in AD, compared with other neurodegenerative and ischemic lesions [285]	93 Individuals with different stages of AD (93), and controls (1024) with cerebral atherosclerosis, Binswanger disease, vascular Parkinsonism.	Brain scintigraphy with TC 99M pertechnetate 555, rheoencephalography (REG), CT and MRI of the brain and cerebral multi-gated angiography (MUGA).	temporal and fronto-parietal areas of all patients with AD, regardless of disease stage: specific changes in cerebral microcirculation which they named dyscirculatory angiopathy of Alzheimer's type (DAAT). DAAT was not found in the controls.
6	Effects of acute hypoxia on cerebral bioenergetics and memory [275]	Recreationally active young males (12)	7 h of either normoxia (fraction of inspired O_2 = 0.21) or hypoxia (fraction of inspired O_2 = 0.12) Cognitive function assessed at baseline (0 h), 2, 4 and 6 h; cerebrovascular and cardiopulmonary monitoring	In hypoxia, oxygen delivery was reduced in middle cerebral artery during central executive tasks and in posterior cerebral artery during memorization and recall; no effect on cerebral blood flow
7	Regional cerebral microvascular perfusion in acute and prolonged hypoxia [286]	Healthy young males (13)	2H or 10h in normobaric normoxia (21% O_2) or normobaric hypoxia (12% O_2); Brain MRI	2 h of hypoxia: perfusion increased frontal cortex-decreased in 'default mode' network; After 10 h decreased blood flow in default mode network more pronounced and widespread, hence reduced local perfusion; Showed relate to vasoconstriction related to vasoconstriction

8	Effects of brain ischaemia on succinate and other metabolites [287]	Normal human brain removed during neurosurgery, mouse brain; mouse model of ischemic stroke induced by transient occlusion of the middle cerebral artery (45 min) followed by reperfusion	human and mouse brain exposed to warm ischemia ex vivo, quantitative and untargeted high-resolution analyses; mass spectrometry imaging (MSI) of stroke model.	warm ischemia ex vivo: time-dependent accumulation of succinate, other significant changes included increases in purine degradation, PUFAs, 5-oxoproline, decreases in adenosine, acylcarnitines<br>Stroke model: succinate accumulated, other TCA metabolites decreased, Dramatic decrease in ATP
9	Association of regional cerebral perfusion in AD with Tau and amyloid [261]	Cognitively normal individuals (84), amnestic MCI (51) AD dementia (21)	Assessed local associations of CBF (arterial spin labelling-MRI), Tau (AV1451-PET and amyloid (AV45/FBB-PET)	Tau-PET was associated with lower CBF in the entorhinal cortex, persisted after excluding AD dementia group, was independent of Aβ. APOE genotype and MRI markers for small vessel disease. Amyloid-PET was associated with lower CBF in temporo-parietal regions
10	Tau deposition in entorhinal cortex related to hypoperfusion [4]	Cognitively normal individuals (20), amnestic MCI (23) AD dementia (1)	baseline [^{18}F] flortaucipir tau PET within 1 year of the ASL MRI and follow-up [^{18}F] flortaucipir tau PET at 6 years	baseline CBF was associated with tau deposition at the 6-year follow-up in the left but not the right entorhinal cortex; findings suggest that a reduction in CBF at the entorhinal cortex precedes tau deposition.
11	Longitudinal changes in CBF in the older hypertensive brain [280]	14 treated hypertensive participants and14 age-matched healthy controls	Changes in regional CBF (r CBF) assessed from PET scans at 1,3,5,7y.	Relative to controls, in the hypertensive group rCBF decreased in prefrontal, anterior cingulate, and occipital areas over time
	**Animal studies**			
12	Neurovascular coupling in the hippocampus and visual cortex [274]	Mice with an implanted cranial window	Neural stimulation of hippocampus and visual cortex; Flow by two photon imaging, doppler flow, haemoglobin spectroscopy, calculated blood oxygen-dependent signal	Compared with visual cortex: hippocampal arteries blunted response: fewer, smaller, dilations. ATP production restricted in tissues furthest from capillaries
13	Identification of leukotrienes C4 and D4 in gerbil brains after ischemia and reperfusion [289]	gerbil forebrains after bilateral common carotid occlusion and reperfusion.	Immunoreactivity of leukotrienes C4 and D4 (potent vasoconstrictors)	Significant increases at 5,10, or 15 min of ischaemia, more marked on reperfusion; highest in forebrain grey matter, undetectable in brain regions remote from ischemic zone
14	Biochemical response to hypobaric oxygen: hippocampus, cortex, cerebellum [290]	Rats exposed to hypobaric oxygen equivalent to 25,000 ft for 0, 3, 7, or 14 days.	Biochemical evaluation of oxidative stress markers and antioxidant status of in each brain region.	Compared with controls, increased lactate dehydrogenase, free radical generation, lipid peroxidation, glutamate dehydrogenase activity, vesicular glutamate transporter expression decreased glutathione reductase, superoxide dismutase activity, reduced glutathione with increased oxidized glutathione.

15	Hippocampal morphology following hypobaric hypoxia [291]	Rats were exposed to hypobaric oxygen for 0 or 4 days at altitudes equivalent to 3500m or 6400m, with assessments 72h and 144h post-6400m exposure.	Hippocampal morphology	Significant cell degeneration and death only in the CA3 region; damage more noticeable with longer time following exposure
16	Oxidative stress in rat brain in hypobaric hypoxia [292]	Rats exposed to hypobaric oxygen equivalent to 6100m for 0, 3, or 7 days.	Evaluation of stress markers in cortex, hippocampus and striatum	Significant increase in free radical production, nitric, lipid peroxidation lactate dehydrogenase greater at 7 days than 3 days; reduced glutathione, glutathione peroxidase, glutathione reductase, superoxide dismutase and reduced/oxidized glutathione. Hippocampus most susceptible.
17	Effect of acute hypobaric hypoxia on SOD and MDA, and mRNA expression of VEGF and HIF1-α in rat brain [293]	Rats were exposed to hypobaric oxygen equivalent to 7000 meters for durations of 0, 1, 2, or 3 days.	SOD and MDA-commercial kits, VEGF and HIF1-α - qRT-PCR.	Increased expression of HIF1-α and VEGF days1,2,3; significant increased MDA, decreased SOD
